# Adipose Tissue–Central Nervous System Axis in Obesity: Molecular Mechanisms, Inflammation, and Nutritional and Technological Implications

**DOI:** 10.3390/metabo16080533

**Published:** 2026-07-29

**Authors:** Serena Castelli, Gilda Aiello, Alessandra De Bruno, Deborah Fratantonio, Gianluca Tripodi, Vincenzo Aiello, Mauro Lombardo, Sara Baldelli

**Affiliations:** 1 Department for the Promotion of Human Science and Quality of Life, San Raffaele Open University, Rome, Via di Val Cannuta, 247, 00166 Rome, Italy; serena.castelli@uniroma5.it (S.C.); gilda.aiello@uniroma5.it (G.A.); alessandra.debruno@uniroma5.it (A.D.B.); vincenzo.aiello@uniroma5.it (V.A.); mauro.lombardo@uniroma5.it (M.L.); sara.baldelli@uniroma5.it (S.B.); 2 IRCCS San Raffaele Roma, 00166 Rome, Italy; 3 Department of Medicine and Surgery, LUM University, S.S. 100 Km 18, 70100 Casamassima, Italy; fratantonio@lum.it

**Keywords:** obesity, adipose tissue–brain axis, neuroendocrine regulation, metabolic inflammation, nutritional modulation

## Abstract

**Highlights:**

**What are the main findings?**

- The adipose tissue–CNS axis is a complex bidirectional network where adipokines, lipid mediators, metabolites, and neural circuits regulate energy balance, metabolism, and inflammation.
- Obesity disrupts this axis through chronic inflammation, neuroinflammation, oxidative stress, and altered lipid signaling, leading to central insulin/leptin resistance and metabolic dysfunction.

**What are the implications of the main findings?**

- Targeting nutrient-derived signals (e.g., fatty acids, SCFAs, polyphenols) and adipose–brain communication pathways offers promising strategies for preventing and treating obesity-related metabolic and neurological disorders.
- Innovative nutritional approaches and food technologies (e.g., functional foods, microbiota modulation, encapsulation systems) may improve metabolic resilience by restoring adipose–CNS axis homeostasis.

**Abstract:**

Obesity is a multifactorial condition characterized by profound metabolic and inflammatory dysregulation that alters the adipose tissue-central nervous system (CNS) relationship. This paper critically summarizes the biochemical and cellular mechanisms governing this bidirectional crosstalk, moving beyond a descriptive perspective to propose an integrated model of peripheral-central interaction. White adipose tissue (WAT) and brown adipose tissue (BAT), modulated by the sympathetic nervous system (SNS), communicate with hypothalamic circuits (POMC and AgRP neurons) both through traditional endocrine signals (leptin, adiponectin, resistin, apelin) and through lipid mediators and extracellular vesicles (EVs) capable of crossing the blood–brain barrier (BBB). Under conditions of nutritional excess, the accumulation of lipotoxic lipid species (such as palmitate and ceramides) and the inflammatory polarization of brain macrophages (M1/BAMs) induce mitochondrial stress and central resistance to leptin and insulin, altering the adiposity set point. In parallel, we review emerging nutritional strategies based on polyunsaturated fatty acids (PUFAs), polyphenols, and short-chain fatty acids (SCFAs), and critically evaluate innovative technological platforms (nanoencapsulation, precision fermentation, and 3D food printing) designed to optimize nutrient bioaccessibility and restore adipose-CNS axis homeostasis, discussing current challenges for clinical translation.

## 1. Introduction

Obesity is one of the most pressing public health challenges worldwide, with prevalence rates continuing to increase across all age groups. According to the World Health Organization, overweight and obesity affect billions of individuals globally and represent major risk factors for type 2 diabetes mellitus, cardiovascular diseases, non-alcoholic fatty liver disease, certain cancers, and neurodegenerative disorders. Beyond excessive fat accumulation, obesity is currently recognized as a complex, multifactorial, and chronic disease characterized by profound alterations in metabolic, endocrine, inflammatory, and neural pathways that collectively disrupt whole-body homeostasis [1,2].

Over the last decades, adipose tissue has emerged as a highly active endocrine and immunometabolic organ rather than a passive energy reservoir. Through the secretion of adipokines, cytokines, extracellular vesicles, lipid mediators, and metabolites, adipose tissue continuously communicates with peripheral organs and the central nervous system (CNS), contributing to the regulation of appetite, energy expenditure, glucose homeostasis, thermogenesis, and behavioral responses [3,4]. In parallel, the CNS integrates nutritional, hormonal, and inflammatory signals to orchestrate adaptive responses through autonomic, neuroendocrine, and behavioral mechanisms [5,6]. This bidirectional communication network, commonly referred to as the adipose tissue–brain axis, plays a pivotal role in maintaining metabolic balance.

In obesity, chronic nutrient excess and adipose tissue expansion profoundly alter this communication system. Adipocyte hypertrophy, immune cell infiltration, and increased production of inflammatory mediators promote a state of chronic low-grade inflammation that extends beyond adipose depots and affects multiple organs, including the brain [1]. At the central level, obesity is associated with hypothalamic inflammation, oxidative stress, mitochondrial dysfunction, altered nutrient sensing, and impaired responsiveness to key metabolic hormones such as leptin and insulin [7]. These alterations contribute to the disruption of neuronal circuits controlling feeding behavior and energy homeostasis, thereby reinforcing metabolic dysfunction and creating a self-perpetuating pathological cycle.

Recent advances in molecular biology, metabolomics, neuroimaging, and nutritional sciences have considerably expanded our understanding of the mechanisms underlying adipose tissue–CNS communication. Particular attention has been directed toward adipokines, bioactive lipids, nutrient-derived metabolites, mitochondrial bioenergetics, and inflammatory pathways that mediate the reciprocal interactions between peripheral metabolic tissues and central neural networks [8,9]. At the same time, emerging evidence suggests that dietary interventions, bioactive compounds, microbiota-derived metabolites, and innovative technological approaches may represent promising strategies to restore the integrity of this axis and improve metabolic health.

Previous reviews have predominantly examined individual components of adipose tissue–brain communication, including adipokine signaling, hypothalamic inflammation, autonomic regulation, gut microbiota-derived metabolites, or nutritional interventions. Although these studies have provided essential mechanistic insights, the different levels of communication are frequently considered as parallel processes rather than as functionally interconnected stages of the same biological system. The distinctive perspective of this review is to conceptualize the adipose tissue–CNS axis as a multilevel metabolic information-processing network. Within this framework, the metabolic and mitochondrial state of adipose tissue determines the generation and molecular composition of adipokines, lipid mediators, metabolites, and extracellular vesicles. These signals are transmitted to the CNS through circulating, blood–brain barrier-dependent, microbial, and neural routes and are subsequently decoded by hypothalamic neurons, glial cells, and nutrient-sensing pathways. The CNS, in turn, modifies adipose tissue metabolism through autonomic and neuroendocrine feedback. We therefore interpret obesity as a failure of bidirectional metabolic information transfer, in which altered peripheral signal generation coexists with defective central signal decoding. By positioning dietary patterns, food matrix properties, microbiota-derived metabolites, and innovative food technologies at specific levels of this communication network, the review further connects molecular pathophysiology with nutritional and technological intervention strategies. Throughout this review, mechanisms supported by consistent experimental and clinical evidence are distinguished from emerging concepts that remain largely based on preclinical observations. When discussing novel signaling pathways or nutritional strategies, the strength of the available evidence is explicitly considered to facilitate a balanced interpretation of current knowledge and future research directions.

## 2. Methods

Relevant studies included in this narrative review were identified through PubMed together with manual screening of reference lists. The search was updated through June 2026, with no lower date restriction; however, priority was given to the most recent studies directly related to adipose tissue–CNS communication and its metabolic, inflammatory, nutritional, or technological modulation. Studies summarized in Tables were selected based on their relevance to adipose tissue–CNS communication, obesity-related metabolic regulation, inflammation, nutritional interventions, and translational significance. Priority was given to randomized controlled trials, mechanistic investigations, recent publications, and landmark studies that substantially contributed to the understanding of the adipose tissue–brain axis.

## 3. Neural and Endocrine Architecture of Adipose Tissue–CNS Communication

The central nervous system (CNS) is the body’s primary integration center for metabolic signals, coordinating appetite, energy expenditure, metabolism, and behavior to maintain homeostasis. In this context, the hypothalamus plays a key role, particularly through the arcuate nucleus, where neurons expressing pro-opiomelanocortin (POMC) and agouti-related peptide (AgRP) of the melanocortin system translate peripheral signals into adaptive responses. In parallel, adipose tissue emerges as a dynamic endocrine organ, capable not only of storing energy but also of actively communicating with the brain via adipokines, bioactive lipids, and inflammatory mediators. The distinct functions of white and brown adipose tissue (WAT and BAT) contribute in a complementary manner to the control of energy balance and thermogenesis. Taken together, these findings highlight the existence of a finely tuned, bidirectional crosstalk between the CNS and adipose tissue, essential for metabolic adaptation and systemic health.

Adipose tissue is a direct target of sympathetic innervation, which finely regulates its metabolic and adaptive functions. In WAT, the terminals of the sympathetic nervous system establish direct control over adipocytes: the local release of norepinephrine (NE) and its interaction with β-adrenergic receptors activate a cascade of intracellular signals that culminates in increased lipolysis and the release of free fatty acids (FFA) and glycerol, essential for the body’s energy supply [10]. The presence and functionality of this innervation have been extensively demonstrated through electrophysiological approaches, sympathectomy procedures, and selective electrical stimulation of nerve fibers, confirming the central role of the sympathetic nervous system (SNS) in regulating lipid metabolism in WAT [11].

The organization of sympathetic projections to the WAT has been elucidated through transsynaptic retrograde neuronal tracing studies. Use of the pseudorabies virus identified a distributed neural network along the spinal cord, brainstem, midbrain, and forebrain, including numerous hypothalamic nuclei, as the source of sympathetic innervation to the WAT [12]. Subsequent studies using neurotropic viruses further refined this framework, showing that hypothalamic projections from the arcuate nucleus (ARC) to the WAT derive predominantly from POMC neurons, while direct contributions from AgRP neurons are absent [13]. It is noteworthy that WAT is organized into different fat pads, including subcutaneous and visceral fat pads, each characterized by specific innervation density, different levels of receptor expression, and distinct sympathetic activity [14,15]. Furthermore, sex-related anatomical differences in sympathetic innervation of WAT suggest the existence of metabolically dimorphic regulation of this tissue [13]. Advanced denervation techniques and three-dimensional imaging of the entire fat pad have revealed that almost all presynaptic fibers present in WAT are sympathetic in nature, as indicated by positivity for tyrosine hydroxylase (TH) and synaptophysin [16].

In addition to the acute regulation of lipid mobilization, sympathetic innervation of WAT also contributes substantially to the development and maintenance of adipose tissue, modulating the entire life cycle of pre-adipocytes, from proliferation and differentiation to apoptosis [17,18,19]. The functional importance of this nervous control had already emerged from pioneering studies demonstrating an increase in FFA concentration following electrical nerve stimulation of isolated fat pads [20], an observation subsequently confirmed in numerous animal models, in which surgical or chemical denervation of WAT is associated with a marked reduction in lipid mobilization [21].

An even more evident role of sympathetic innervation emerges in BAT, where SNS activity represents an essential prerequisite for the activation of thermogenesis. Sympathetic denervation studies conducted in rodents, together with pharmacological interventions based on β3-adrenergic receptor agonists and antagonists, have demonstrated that noradrenergic stimulation is indispensable for the activation of the thermogenic function of BAT [22,23]. In support of this concept, recent experiments have shown that electrical stimulation applied to the dorsal surface of interscapular BAT induces a significant increase in tissue temperature without modifying core body temperature; this effect is completely abolished already two days after sympathetic denervation [24]. The absence of changes in UCP1 content and the thermogenic response to norepinephrine in this phase excludes an intrinsic loss of BAT thermogenic capacity, instead indicating the requirement of local nervous release of NE for tissue activation [24]. Pharmacological studies have further confirmed that this effect is mediated by β-adrenergic receptors, strengthening the central role of the SNS in the control of brown thermogenesis.

The functional dependence of BAT on sympathetic innervation has been validated by both surgical and chemical denervation approaches, consolidating the idea that the SNS represents a key regulator of systemic energy homeostasis [25]. Furthermore, sympathetic nerve fibers are present within the adipose parenchyma and increase in density in response to cold exposure, a condition that stimulates sympathetic outflow and promotes adipose tissue activation. This remodeling of parenchymal innervation, observed in both WAT and BAT, is required for the induction of thermogenic gene expression in response to cold [26]. Consistently, selective blockade of sympathetic activity in these tissues impairs cold-induced thermogenesis [27,28]. Finally, recent evidence has shown that localized adipose tissue sympathectomy reduces thermogenesis and predisposes to the development of obesity in murine models, confirming the crucial role of adipose sympathetic drive in the control of systemic metabolism [25].

As discussed, the SNS is a key regulator of lipolysis in WAT, primarily through NE release and subsequent activation of β-adrenergic receptors (βARs; β1–β3 subtypes) expressed on adipocytes. βAR activation triggers the recruitment of Gsα, stimulating adenylate cyclase and resulting in increased intracellular levels of cyclic AMP (cAMP). This, in turn, enhances the activity of protein kinase A (PKA), which phosphorylates and activates two key components of the lipolytic machinery: hormone-sensitive lipase (HSL) and the lipid droplet coat protein perilipin A [29]. Under physiological conditions characterized by increased energy demand, such as stress or exercise, increased NE release from sympathetic endings amplifies βAR activation, promoting the mobilization of FFA from WAT [30].

Central control of this axis is further evidenced by the CNS’s ability to modulate βAR expression in WAT. Specifically, central infusion of melanin-concentrating hormone (MCH), acting on ARC neurons that express the MCH receptor, reduces βAR expression in white adipose tissue, attenuating its lipolytic response [31]. In parallel, the main hypothalamic circuits involved in energy balance, consisting of POMC and AgRP neurons, exert a direct control over WAT lipolysis. Selective activation of AgRP neurons has been associated with a reduction in circulating fatty acid levels and a modulation of HSL activity in WAT through SNS-dependent pathways [32]. Conversely, chemogenetic manipulation of POMC neurons suppresses the lipolytic molecular program and reduces fasting-induced lipid mobilization [33].

Among the endocrine signals modulating adipose sympathetic activity, leptin plays a central role. This adipokine, known for its involvement in the control of energy balance, promotes lipolysis in WAT through the activation of sympathetic neurons innervating adipocytes, an effect partly mediated by βAR activation [34]. Centrally, leptin acts on its receptors expressed by AgRP and POMC neurons of the ARC, regulating sympathetic innervation of adipose tissue through a descending neuronal pathway that depends on the production of brain-derived neurotrophic factor (BDNF) by neurons in the paraventricular nucleus of the hypothalamus (PVH) [35]. However, leptin also exerts a complementary anti-lipogenic effect, mediated by hypothalamic phosphatidylinositol 3-kinase signaling and the endocannabinoid system, particularly anandamide, in WAT [36]. In stark contrast to leptin, insulin infusion into the mediobasal hypothalamus (MBH) reduces SNS activity, promotes the expression of lipogenic proteins in WAT, and inhibits HSL activity, resulting in suppression of lipolysis [37].

An additional level of central regulation involves the control of leptin gene expression in WAT, which is also dependent on sympathetic innervation. Lesions of the suprachiasmatic nucleus (SCN), responsible for maintaining circadian rhythms, have been shown to abolish the daily rhythmicity of leptin secretion, highlighting a direct link between the biological clock, the SNS, and adipose function [38]. In addition to hormonal and metabolic regulation, the SNS also influences adipose tissue development by modulating the proliferation of pre-adipocytes. This has been observed in denervation models and in transgenic mice lacking the neuronal transcription factor Nscl-2 that develop obesity in adulthood in association with an increased number of pre-adipocytes [17,19]. In Nscl-2 mutant mice, reduced sympathetic innervation is also accompanied by a marked decrease in WAT microvasculature, an aspect that could contribute to the structural and functional alterations observed in adipose tissue [19]. Overall, these findings broaden the role of the SNS beyond its classical function of inducing lipolysis, including the control of hormone secretion and adipogenic processes.

In response to environmental stimuli, such as cold exposure, adrenaline-mediated activation of the SNS promotes the phenotypic conversion of white adipocytes into cells with characteristics similar to those of brown adipose tissue, a process known as “browning.” During this adaptation, white adipocytes acquire a thermogenic function, dissipating energy in the form of heat [39]. Browning of WAT results from the activation of multiple signaling pathways that converge on the induction of the expression of the uncoupling protein UCP1 and other BAT-specific genes, including PPARγ, PRDM-16, and PGC-1ɑ [40]. The classical mechanism underlying this process involves SNS excitation and activation of the β3-adrenergic receptor signaling pathway, with PRDM-16 acting as a key regulator of the thermogenic transcriptional program [41,42]. More recent studies have also highlighted a significant role for thyroid hormones in promoting WAT browning, suggesting the existence of alternative or complementary mechanisms to sympathetic activation [43]. Taken together, these observations underscore how sympathetic innervation represents a crucial element in maintaining the metabolic health of adipose tissue, acting at functional, developmental, and cell fate determination levels.

### Role of Adipokines in the Adipose–Brain Axis

Maintaining the body’s energy balance depends on a complex and highly integrated network of molecular signals and communication pathways operating at multiple levels [44,45]. In this context, the CNS plays a key role, as it is constantly exposed to peripheral information transmitted both through neural circuits and humoral signals, such as hormones and circulating nutrients, which reflect the organism’s overall energy status [5]. Central processing of these inputs allows the CNS to generate targeted efferent responses, capable of modulating metabolic fluxes in the main tissues involved in energy storage and mobilization, including the liver, adipose tissue, and skeletal muscle [46], as well as influencing feeding behavior and food intake [6].

Within the CNS, specific brain areas show particular sensitivity to different aspects of energy balance. Regions such as the hypothalamus and brainstem respond predominantly to homeostatic variations related to caloric availability, while structures such as the striatum, nucleus accumbens, and ventral tegmental area are more involved in evaluating the hedonic aspects of food, including palatability and reward mechanisms [5,6,47].

In recent decades, starting with the identification of leptin in 1995, the endocrine nature of adipose tissue has become increasingly clear. In addition to its classical function as an energy store and source of metabolic substrates, adipose tissue and adipocytes are now recognized as active endocrine cells, capable of synthesizing and releasing a wide range of signaling molecules [3,4,48].

Among these, adipokines—a heterogeneous set that includes hormones, cytokines, extracellular matrix (ECM) components, growth factors, and vasoactive molecules—represent key mediators of communication between adipose tissue and the brain. Through their action on the CNS and on the areas responsible for controlling energy balance, adipokines contribute significantly to the regulation of appetite, metabolism and overall energy homeostasis, outlining a functional adipose tissue-brain axis.

First of all, we recall adiponectin, also known as Acrp30 or AdipoQ. Adiponectin is a pleiotropic adipokine produced predominantly by adipose tissue [49] and is one of the most abundant molecules circulating in human plasma, with concentrations ranging from 2 to 20 μg mL^−1^. In recent years, this adipokine has been recognized as a key element in the complex signaling network that coordinates the body’s energy balance. The broad spectrum of biological actions attributed to adiponectin is supported both by the presence of different molecular isoforms and by the tissue- and cell-specific distribution of its receptors.

Adiponectin exerts its effects through two membrane receptors, AdipoR1 and AdipoR2 [50], both widely expressed in peripheral tissues, including skeletal muscle, liver, and adipose tissue [51]. However, their expression is not limited to the periphery, as both receptors have also been identified in the central nervous system (CNS). In particular, AdipoR1 and AdipoR2 are present in several brain regions involved in metabolic control, including the cerebral cortex, hypothalamus, pituitary gland, brainstem, and hippocampus [52]. The distribution of adiponectin receptors in these areas suggests a direct involvement of the adipokine in the central modulation of energy homeostasis.

The possibility that adiponectin acts directly on the CNS is supported by experimental evidence indicating its ability to cross the blood–brain barrier. In particular, the low molecular weight trimeric form of adiponectin has been detected in the cerebrospinal fluid following intravenous administration in mouse models lacking endogenous adiponectin [53]. Functional studies have demonstrated that adiponectin participates in the regulation of food intake and energy homeostasis in coordination with leptin [54]. In this context, adiponectin is able to modulate the expression of hypothalamic neuropeptides, reducing orexigenic signals, such as neuropeptide Y (NPY), and promoting anorexigenic ones, including melanocortin (αMSH). However, conflicting data indicate that, under certain conditions, adiponectin can also stimulate food intake through the activation of AMP-activated protein kinase (AMPK) at the hypothalamic level, suggesting a contextual and metabolic-dependent role [55].

In addition to appetite control, the central action of adiponectin contributes significantly to the improvement of insulin sensitivity and glucose tolerance, as demonstrated in mouse models of obesity [56]. At the CNS level, adiponectin interacts with key insulin signaling pathways, promoting improved global metabolic function. This integration between adiponectin and insulin signaling is crucial in maintaining energy homeostasis and preventing metabolic dysfunction.

Adiponectin also exerts neuroprotective effects, with potential implications for the prevention of cognitive decline and neurodegenerative diseases [57]. Its anti-inflammatory and antioxidant properties help counteract microglial activation and brain inflammation induced by high-fat diets [58]. These effects further strengthen adiponectin’s role as a key modulator of the interplay between metabolism and neuronal function, extending its relevance beyond energy control to neurodegenerative processes [59].

Overall, a thorough understanding of the mechanisms through which adiponectin acts on the CNS provides important insights into the pathophysiology of obesity-associated metabolic disorders. Therapeutic approaches aimed at restoring or enhancing adiponectin signaling could therefore represent a promising strategy to improve metabolic health and reduce complications associated with excess weight.

Apelin has also emerged as an important regulator within the circuits governing energy balance in the organism [60]. This bioactive peptide acts as an endogenous ligand for the APJ receptor, a G-protein-coupled receptor, and is present in several biologically active forms, including isoforms consisting of 13, 17, or 36 amino acids, all derived from a common 77-amino acid precursor protein [61]. Apelin is produced and released by numerous tissues, including adipose tissue, and growing evidence indicates that it participates in the neural regulation of energy balance, modulating processes such as appetite, metabolism, and global energy homeostasis [60].

The apelin/APJ system has a broad tissue distribution, including key brain structures such as the hypothalamus. In this context, acute central administration of apelin-13 in rats has been shown to increase food intake, an effect attributed to a partial inhibition of serotonin synthesis and release, associated with an increase in orexin A gene expression [62]. In line with these findings, subsequent studies have shown that apelin administration, both centrally and peripherally, is able to modulate food intake and to influence insulin sensitivity in murine models [63]. Furthermore, chronic central infusion of apelin has been observed to determine a reduction in overall energy expenditure and thermogenic activity in mice, suggesting a relevant role of this peptide in the long-term control of energy metabolism [64]. Resistin was initially described as an adipokine related to insulin resistance and obesity, but subsequently emerged as a relevant modulator of energy homeostasis [65]. Belonging to the resistin-like molecules (RELM) family, which also includes RELM-α and RELM-β, it presents species-specific differences in its cellular origin: in rodents it is secreted predominantly by white adipose tissue, with levels varying according to sex and adipose depot, while in humans its expression in adipocytes is modest and macrophages represent the main source of production [66]. In conditions of obesity, increased macrophage infiltration into visceral adipose tissue constitutes the main source of resistin [67].

Although the specific receptor has not been definitively clarified, experimental evidence indicates that resistin may exert its effects through binding to the Toll-like receptor 4 (TLR4), activating pro-inflammatory signaling pathways [68]. The presence of TLR4 in brain areas involved in the control of energy balance suggests a direct interaction with the central nervous system. Indeed, central administration of resistin is associated with increased sympathetic activity, development of hypertension, and worsening insulin sensitivity via TLR4-dependent mechanisms [69]. Considering that obesity is characterized by sympathetic hyperactivation, chronic low-grade inflammation, and increased cardiovascular risk, resistin could represent a key element in the pathogenesis of these alterations [70].

Further evidence indicates that resistin interferes with other central metabolic signals: its intracerebral administration reduces the hypothalamic expression of adiponectin receptors (AdipoR1/R2) and contributes to hepatic insulin resistance [71]. In addition to its metabolic role, resistin possesses marked pro-inflammatory properties; since chronic neuroinflammation alters signal transduction in the CNS, resistin-mediated activation of inflammatory circuits could compromise neuronal communication implicated in energy control [69].

In light of its involvement in the neural regulation of metabolism and its association with cardiometabolic dysfunction, resistin emerges as a potential therapeutic target. However, further studies are needed to clarify the feasibility and efficacy of strategies aimed at selectively modulating its signaling pathways in the treatment of obesity-related complications.

Having defined the neural and endocrine routes that constitute the adipose tissue–CNS axis, the following section examines how obesity disrupts these communication channels through inflammation, oxidative stress, and cellular metabolic dysfunction.

**Figure 1:**
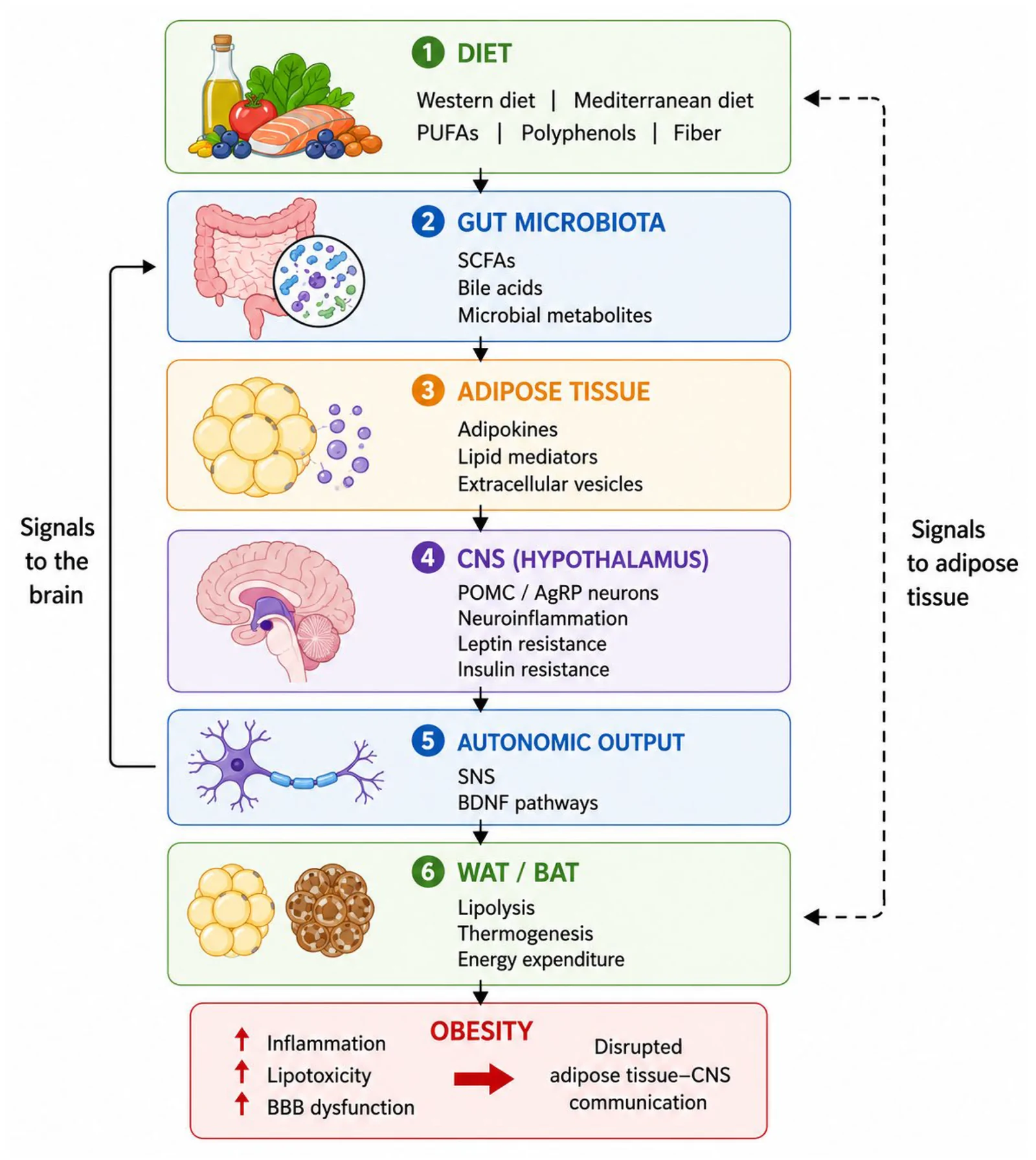
Simplified integrated model of bidirectional adipose tissue–CNS communication in obesity. Dietary patterns and food-derived bioactive compounds influence gut microbiota composition and activity, leading to the production of metabolites such as short-chain fatty acids (SCFAs), bile acids, and other microbial-derived signaling molecules. These mediators modulate adipose tissue function, affecting the secretion of adipokines, lipid mediators, and extracellular vesicles that convey metabolic information to the central nervous system (CNS). Within the hypothalamus, nutrient sensing, neuroinflammation, leptin resistance, and insulin resistance alter the activity of key neuronal populations, including pro-opiomelanocortin (POMC) and agouti-related peptide (AgRP) neurons. The CNS subsequently regulates adipose tissue metabolism through autonomic and neuroendocrine pathways, including sympathetic nervous system (SNS)-mediated control of lipolysis, thermogenesis, and energy expenditure in white and brown adipose tissue (WAT and BAT). Obesity disrupts this bidirectional communication network through chronic inflammation, lipotoxicity, and blood–brain barrier (BBB) dysfunction, promoting progressive metabolic dysregulation.

The complex interactions described throughout this review are summarized in Figure 1, which illustrates the bidirectional communication network linking dietary factors, gut microbiota, adipose tissue signaling, hypothalamic regulation, and autonomic control of energy homeostasis.

## 4. Neural Metabolic Alterations in Obesity

There is substantial scientific evidence documenting neuronal alterations associated with overweight and obesity. Obesity is a systemic metabolic disease that affects multiple organs, generating a state of chronic low-grade systemic inflammation. This persistent inflammatory milieu is characterized by increased circulating cytokines, altered adipokine secretion, insulin resistance, and metabolic dysregulation, all of which can influence brain structure and function.

At the CNS level, obesity has been associated with both functional and structural changes. Neuroimaging studies have reported alterations in gray matter volume, white matter integrity, and connectivity patterns, particularly in regions involved in appetite regulation, reward processing, executive function, and memory, such as the hypothalamus, hippocampus, and prefrontal cortex. Some of these alterations are attributable to obesity-associated neuroinflammation, whereas others still lack a clearly defined etiopathogenesis [72].

### 4.1. Oxidative Stress and Neuroinflammation in Obesity

**Figure 2:**
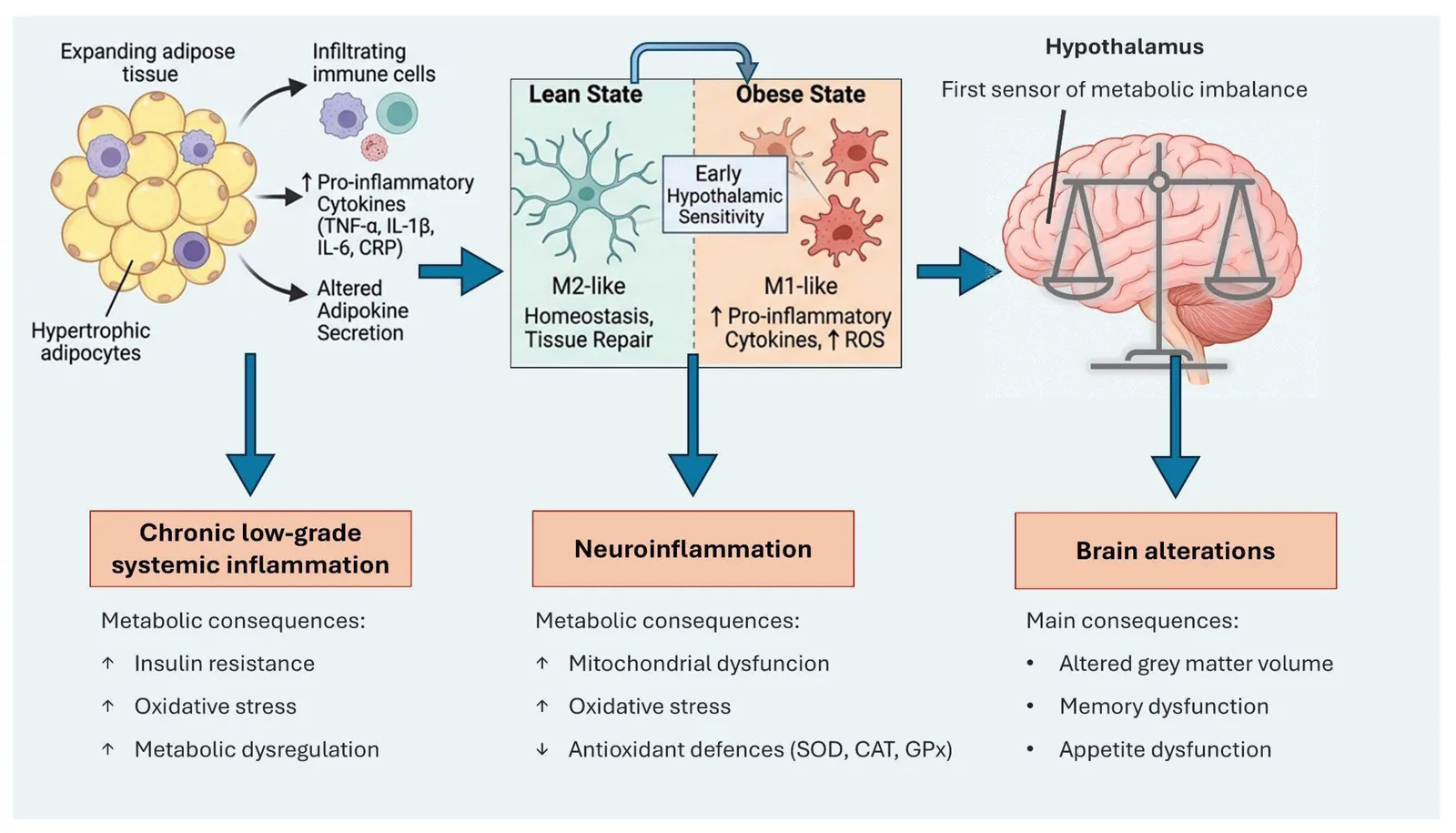
Obesity promotes adipose tissue expansion and immune cell infiltration, leading to chronic low-grade systemic inflammation characterized by increased pro-inflammatory cytokines and altered adipokine secretion. These signals trigger neuroinflammation through the activation and polarization of brain macrophages and microglia toward a pro-inflammatory phenotype. The hypothalamus, as the primary sensor of metabolic imbalance, is particularly susceptible to these alterations. Obesity-associated neuroinflammation is accompanied by oxidative stress, mitochondrial dysfunction, and impaired antioxidant defenses, ultimately contributing to structural and functional brain alterations, including impaired memory, dysregulated appetite control, and changes in gray matter volume.

In obesity, the expansion of adipose tissue represents a major driver of systemic inflammation. Both hypertrophic adipocytes and immune cells infiltrating the adipose tissue, particularly macrophages and lymphocytes, actively contribute to the establishment of a chronic proinflammatory milieu (Figure 2). This condition is characterized by increased production and release of inflammatory mediators, including tumor necrosis factor-α (TNF-α), plasminogen activator inhibitor-1 (PAI-1), C-reactive protein (CRP), interleukin-1β (IL-1β), and interleukin-6 (IL-6) [1]. Chronic low-grade systemic inflammation represents not only a hallmark of obesity, but also a key pathophysiological mechanism linking excess adiposity to brain dysfunction. This persistent inflammatory state contributes to altered neuroendocrine signaling, impaired metabolic sensing, and progressive neuronal stress, ultimately affecting central regulation of energy balance and behavior [2].

Adipose tissue-derived inflammatory signals can influence the CNS through multiple and complementary routes. Cytokines, adipokines, free fatty acids, and extracellular vesicles released by hypertrophic adipocytes and infiltrating immune cells enter the systemic circulation and may reach or signal across the blood–brain barrier (BBB). Rather than freely diffusing into the brain, these mediators can interact with receptors and transport systems expressed by brain endothelial cells, activate perivascular and border-associated macrophages, or increase BBB permeability by altering endothelial tight junctions. Circulating signals may also access brain regions characterized by a more permissive vascular interface, including the median eminence adjacent to the arcuate nucleus. These mechanisms promote chemokine production, microglial activation, and local inflammatory amplification, thereby connecting peripheral adipose tissue inflammation with central neuroinflammation.

Importantly, neuroinflammation appears to be an early and initiating event in obesity-related brain alterations, often preceding overt weight gain and the establishment of peripheral metabolic complications. Experimental and preclinical studies have demonstrated that inflammatory responses within the CNS can be rapidly induced by nutritional excess. For example, signs of neuroinflammation have been detected in the brain after as little as one day of exposure to a high-fat diet, suggesting that dietary lipids themselves can act as potent inflammatory stimuli [73].

Among brain regions, the hypothalamus is particularly susceptible to circulating metabolic and inflammatory signals because it contains specialized vascular interfaces and is located in close proximity to the median eminence, where the BBB is relatively more permissive. This anatomical organization facilitates nutrient and hormone sensing but may also increase hypothalamic exposure to obesity-associated cytokines, adipokines, and lipid mediators. Consequently, hypothalamic neuroinflammation is characterized by early activation of microglia and border-associated macrophages, cytokine production, and cellular stress responses that impair neuronal circuits controlling appetite, satiety, glucose homeostasis, and autonomic output [7]. These early inflammatory changes may therefore play a causal role in the dysregulation of energy balance, creating a vicious cycle that promotes further weight gain and metabolic deterioration [74].

Macrophages are highly plastic immune cells that modulate inflammation according to their activation and differentiation state. Classically activated macrophages (M1 phenotype) promote the initiation and amplification of the inflammatory response through the secretion of proinflammatory cytokines and the generation of reactive oxygen species (ROS). In contrast, alternatively activated macrophages (M2 phenotype) are primarily involved in the resolution phase of inflammation, contributing to tissue repair, extracellular matrix remodeling, and the release of growth factors that support regeneration (Figure 2) [75].

Brain macrophages comprise two principal populations: parenchymal microglia and border-associated macrophages (BAMs), which reside in extra-parenchymal compartments such as the meninges, perivascular spaces, and choroid plexus. Together, these cell types constitute the innate immune system of the CNS and are essential for maintaining tissue homeostasis, immune surveillance, and proper neuronal function. Microglia are the resident macrophages of the brain parenchyma and represent the first line of active immune defense within the CNS. They contribute to synaptic pruning, neurodevelopment, and the regulation of neuroplasticity under physiological conditions. Border-associated macrophages, although less abundant within the parenchyma, play a complementary role at CNS interfaces. Positioned strategically at brain borders, they participate in monitoring peripheral signals, regulating leukocyte trafficking, and maintaining blood–brain barrier integrity [75,76]. In healthy, lean individuals, adipose tissue macrophages predominantly exhibit an M2-like phenotype. Under these physiological conditions, they produce minimal amounts of proinflammatory cytokines and express enzymes such as arginase, which limits nitric oxide production and favors polyamine synthesis, thereby supporting tissue homeostasis. However, in the context of obesity, the adipose tissue microenvironment undergoes profound changes characterized by hypoxia, adipocyte hypertrophy, and increased lipid spillover. These alterations promote macrophage recruitment and polarization toward a more proinflammatory phenotype [74].

Beyond immune alterations driven by changes in microglial polarization, neuroinflammation in obesity is also closely linked to endocrine dysregulation, particularly involving the hypothalamic–pituitary–adrenal (HPA) axis [77]. The HPA axis represents a central neuroendocrine system that coordinates the stress response and regulates glucocorticoid secretion. In conditions of chronic metabolic stress, such as obesity, persistent activation of the HPA axis can lead to sustained elevations in circulating glucocorticoids. Although glucocorticoids exert anti-inflammatory effects in acute settings, prolonged exposure may paradoxically promote immune imbalance, impair glucocorticoid receptor sensitivity, and contribute to central inflammatory signaling. In addition, several conditions and comorbidities frequently associated with obesity further exacerbate dysregulation of the HPA axis, including insomnia, depression, and obstructive sleep apnea [78]. These disorders are themselves linked to chronic stress signaling and contribute to sustained activation or maladaptive regulation of the HPA axis. For instance, sleep disturbances significantly alter circadian cortisol rhythms, often leading to elevated evening cortisol levels and a blunted diurnal decline, thereby reinforcing systemic and central inflammatory tone [77]. Moreover, metabolic signals directly interact with neuroendocrine tissues. Insulin, whose circulating levels are typically increased in obesity due to insulin resistance, can act on pituitary and adrenal progenitor cells. Under chronic hyperinsulinemic conditions, these cells may become metabolically primed toward a hyperfunctional state, resulting in enhanced hormone production. This metabolic priming may further amplify glucocorticoid output and disrupt feedback regulation within the HPA axis [77,79].

Another major contributor to neuroinflammation in obesity is oxidative stress. Oxidative stress results from an imbalance between ROS production and the cellular antioxidant defense capacity. Therefore, it is not solely determined by increased ROS generation, but also by an impaired buffering system that fails to neutralize oxidative damage effectively. On one hand, obesity is characterized by mitochondrial dysfunction, which represents a primary intracellular source of excessive ROS [80]. The chronic oversupply of nutrients—particularly fatty acids—leads to enhanced β-oxidation in an attempt to dispose of lipid accumulation. This metabolic overload increases electron flux through the mitochondrial respiratory chain, favoring electron leakage at complexes I and III and promoting superoxide production [81]. Over time, sustained mitochondrial stress impairs oxidative phosphorylation efficiency, further amplifying ROS generation and establishing a self-perpetuating cycle of metabolic dysfunction and oxidative damage. On the other hand, persistent low-grade inflammation in obesity compromises endogenous antioxidant defenses. Chronic exposure to elevated adipokines and proinflammatory cytokines can suppress key antioxidant enzymes such as superoxide dismutase (SOD), catalase (CAT), and glutathione peroxidase (GPx). As a result, the capacity to detoxify superoxide, hydrogen peroxide, and lipid peroxides is reduced, exacerbating redox imbalance. Excessive adipokine signaling enhances NADPH oxidase activity and mitochondrial ROS production, further contributing to oxidative stress [82].

Additionally, adipose tissue expansion is often accompanied by local hypoxia due to insufficient vascularization of hypertrophic adipocytes. Hypoxia activates hypoxia-inducible factors (HIFs), which can stimulate inflammatory and oxidative pathways. Hypoxia-driven mitochondrial alterations and increased ROS generation reinforce systemic oxidative stress, which can propagate to the CNS [83]. In the brain, elevated ROS levels can activate microglia, damage lipids and proteins, impair synaptic function, and disrupt blood–brain barrier integrity. Therefore, obesity-induced oxidative stress plays a central role in amplifying neuroinflammatory processes and contributing to neuronal vulnerability.

### 4.2. Cellular Metabolic Alterations Induced by Nutritional Excess in the CNS

Obesity-associated neuronal alterations can be broadly categorized into macroscopic and microscopic changes. At the macroscopic level, neuroimaging studies have consistently reported global and regional variations in gray matter volume in individuals with obesity. Rather than a uniform reduction, these changes reflect a complex redistribution of gray matter, characterized by volumetric decreases in some brain regions and increases in others [84]. More specifically, reduced gray matter volume has frequently been observed in areas involved in executive control, reward processing, and cognitive regulation of feeding behavior, such as the prefrontal cortex, hippocampus, and certain limbic structures [85]. These reductions have been associated with impaired cognitive flexibility, altered decision-making, and dysregulated appetite control. Conversely, volumetric increases have been described in regions implicated in energy homeostasis and motivational circuitry, including components of the hypothalamus and striatum, potentially reflecting inflammatory swelling, gliosis, or adaptive remodeling processes [86]. Importantly, these macroscopic structural changes are thought to result from a combination of neuroinflammation, altered synaptic plasticity, metabolic stress, vascular dysfunction, and hormonal imbalances.

At the molecular level, obesity profoundly reshapes the brain metabolome, reflecting altered energy handling, redox balance, and neuromodulatory signaling. One of the most consistently reported alterations concerns NAD^+^ homeostasis. Obesity disrupts NAD^+^ levels and related metabolic pathways in the brain in a sex-independent manner, suggesting a fundamental impairment in redox metabolism and mitochondrial function [87]. Given the central role of NAD^+^ in oxidative phosphorylation, sirtuin activity, DNA repair, and cellular stress responses, its dysregulation may critically affect neuronal resilience and metabolic flexibility.

Beyond NAD^+^ metabolism, many obesity-induced metabolic alterations in the brain appear to be sex-specific. In males, the majority of altered metabolites are linked to pathways involved in energy metabolism, lipid handling, and oxidative stress, indicating a pronounced metabolic remodeling. This may reflect differences in substrate utilization, hormonal regulation, or inflammatory responses between sexes. In contrast, in females, obesity has been shown to preferentially affect metabolites involved in neuromodulation and behavior [88].

Many of the metabolic alterations observed at the cellular level in the central nervous system therefore derive from changes in NAD^+^ levels. Genomic integrity in neurons is critically dependent on adequate NAD^+^ availability. NAD^+^ functions as an essential co-substrate for key DNA repair enzymes, including poly(ADP-ribose) polymerases (PARPs), as well as for NAD^+^-dependent deacetylases such as SIRT1 and SIRT6, which regulate chromatin stability, transcriptional responses, and cellular stress adaptation [89].

DNA repair and chromatin regulation are closely interconnected with one-carbon metabolism, which includes the folate–vitamin B12–choline axis. This metabolic pathway generates S-adenosylmethionine (SAM), the universal methyl donor required for DNA and histone methylation reactions. Indeed, one-carbon metabolism plays a pivotal role in supporting methylation reactions and neural epigenetic regulation, which are essential for proper gene expression, synaptic plasticity, and memory consolidation. Reduced efficiency of this pathway, as occurs with obesity-associated micronutrient imbalance or microbiome shifts, can therefore disrupt methyl donor availability and neural methylation patterns, potentially impairing memory-related neural circuits [90]. Advanced metagenomic and metabolomic studies have begun to define how systemic metabolic pathways—including one-carbon metabolism and branched-chain amino acid (BCAA) metabolism—are linked to cognitive function and memory performance, and how these relationships are altered in obesity. In particular, metagenomic analyses have associated variations in one-carbon metabolic pathways and levels of aromatic and branched-chain amino acids with measures of short-term and working memory, as well as with the volume of key brain regions such as the hippocampus and frontal cortex [91]. Indeed, branched-chain amino acids such as leucine, isoleucine, and valine are not only substrates for protein synthesis and energy metabolism but also act as metabolic and signaling modulators within the brain. Altered BCAA metabolism has been linked to changes in neurotransmitter homeostasis and brain energy dynamics; disruptions in BCAA availability, catabolism, or transport may influence excitatory–inhibitory balance, synaptic signaling, and cognitive processing. Advanced metabolomics work has shown that obesity is often accompanied by perturbations in BCAA pathways, which correlate with metabolic dysfunction and insulin resistance [92].

These pieces of evidence make obesity not merely a peripheral metabolic disorder but a systemic condition capable of profoundly reshaping brain structure, signaling, redox balance, epigenetic regulation and neuronal metabolism, ultimately compromising neuronal homeostasis and cognitive function.

These obesity-associated alterations provide the pathological framework for understanding how specific nutrient-derived metabolites and lipid mediators initiate, amplify, or counteract adipose–brain dysfunction. Although experimental models have provided essential mechanistic evidence linking nutritional excess, hypothalamic inflammation, microglial activation, and impaired metabolic signaling, the translation of these findings to humans remains incomplete. Most causal relationships have been established in rodents or cellular models, whereas human evidence is mainly based on neuroimaging, circulating biomarkers, and observational associations. Therefore, the molecular mechanisms described in this section should not be directly interpreted as validated therapeutic targets in humans. Large-scale randomized clinical trials integrating metabolic outcomes with neuroimaging, central inflammatory markers, and functional assessments of hypothalamic signaling are required to determine their clinical relevance. The following sections therefore focus on the nutrient-derived mediators capable of modulating these mechanisms, rather than reiterating their individual molecular functions.

### 4.3. Trans-BBB Transport Dynamics, Spatial Heterogeneity, and Temporal Sequencing of Lipid Signaling

To understand the lipid-mediated communication between adipose tissue and the brain, it is critical to dissect the specific lipid species involved and their mechanisms of transport across the blood–brain barrier (BBB). Ceramides, particularly long-chain species (C16:0 and C18:0), are elevated in circulation during obesity due to increased hepatocyte and adipocyte de novo synthesis [93]. Because of their highly hydrophobic nature, current evidence suggests that circulating ceramides may reach the CNS through lipoprotein-associated transport and extracellular vesicles, although the precise mechanisms remain incompletely characterized. Lysophosphatidic acid (LPA) is a lipid mediator produced by the lysophospholipase D enzyme autotaxin, which is highly expressed in adipocytes [94]. Circulating LPA interacts with specific G-protein coupled receptors (LPAR1–6) present on the luminal side of the brain endothelial cells [95]. LPA signaling through LPAR1 activates the Rho/ROCK pathway, inducing cytoskeletal rearrangements and the disassembly of tight junction proteins (such as claudina-5 and occludin), thereby transiently opening the paracellular pathway and allowing both LPA and inflammatory cytokines to enter the brain parenchyma [96]. Prostaglandins, specifically prostaglandin E2 (PGE2), act as key inflammatory signaling molecules. Under physiological conditions, circulating PGE2 crosses the BBB via organic anion transporters (such as OAT3) and multidrug resistance-associated proteins (such as MRP4). In obesity, the systemic chronic inflammatory state upregulated endothelial cyclooxygenase-2 (COX-2) expression directly at the BBB [97]. This results in the local, endothelial-derived overproduction of PGE2, which is released directly into the hypothalamic parenchyma, triggering neuroinflammation and disrupting POMC neuron excitability. In obesity, the BBB becomes chronically compromised [98]. The continuous exposure of the brain endothelium to high levels of saturated free fatty acids (like palmitate) and TNF-α leads to the chronic downregulation of tight junctions, resulting in a ‘leaky’ BBB. This structural disruption allows an uncontrolled influx of peripheral lipotoxic species and inflammatory cytokines, perpetuating central metabolic resistance.

## 5. Nutrient-Derived and Bioactive Mediators of Adipose Tissue–Brain Communication

Nutrients are not only energetic substrates but also chemically diverse signaling molecules able to regulate cellular and systemic homeostasis. Their biological activity depends on specific structural features, including carbon chain length, degree of saturation, double-bond position, stereochemistry, and polarity, all of which influence membrane incorporation, receptor binding, intracellular trafficking, and metabolic fate. In this framework, lipids represent one of the most heterogeneous classes of biomolecules, comprising free fatty acids, triacylglycerols, phospholipids, sphingolipids, sterols, and oxygenated derivatives, each endowed with distinct signaling functions [9].

Building upon the molecular mechanisms described in the previous sections, the following sections focus on how nutrient-derived mediators influence adipose tissue–CNS communication. Rather than re-describing the biological functions of inflammatory mediators and adipokines, emphasis is placed on the nutritional modulation of these signaling pathways and their potential translational relevance. Polyphenols, in particular, have been widely associated with suppression of NF-κB-driven inflammation and activation of antioxidant pathways such as Nrf2, whereas SCFAs act as metabolic intermediates and receptor ligands in host tissues [99,100,101]. A further relevant aspect is the ability of selected nutrients and metabolites to cross biological barriers, including the blood–brain barrier (BBB). Long-chain fatty acids and specialized lipids do not simply diffuse passively but often require transport systems such as fatty acid transport proteins and fatty acid-binding proteins. For example, FATP1 contributes to brain uptake of docosahexaenoic acid (DHA), supporting the concept that the chemical nature of nutrients strongly determines their access to central metabolic circuits [102].

### 5.1. Lipid-Derived Mediators in Adipose–Brain Signaling

#### 5.1.1. Fatty Acids and Lipid Sensing

Among nutrient-derived signaling molecules, fatty acids play a pivotal role in the bidirectional communication between adipose tissue and the CNS. Saturated fatty acids (SFAs), especially palmitate, are strongly linked to metabolic inflammation and lipotoxicity. At the central level, SFAs readily affect hypothalamic neurons and glial cells, where they activate multiple stress-related pathways, including Toll-like receptor 4 (TLR4)-dependent signaling, nuclear factor-κB (NF-κB) activation, c-Jun N-terminal kinase (JNK) pathways, and endoplasmic reticulum stress. These events promote microglial activation, cytokine production, and defective neuronal signaling, ultimately contributing to central insulin and leptin resistance and to the establishment of hypothalamic dysfunction in obesity [103]. In addition, chronic exposure to SFAs impairs mitochondrial function and autophagic flux in hypothalamic neurons, further exacerbating neuronal stress and disrupting nutrient sensing mechanisms. Thus, excessive SFA signaling represents a critical link between adipose tissue dysfunction and neuroinflammatory processes within central metabolic circuits.

By contrast, unsaturated fatty acids—particularly omega-3 polyunsaturated fatty acids (PUFAs) such as eicosapentaenoic acid (EPA) and docosahexaenoic acid (DHA)—generally exert anti-inflammatory, insulin-sensitizing, and neuroprotective effects. In hypothalamic neurons and glial cells, DHA can activate the G protein–coupled receptor GPR120 (also known as FFAR4), leading to the suppression of inflammatory signaling pathways and attenuation of cytokine production. Through these mechanisms, omega-3 PUFAs counterbalance the detrimental effects of saturated fatty acids and contribute to the maintenance of central metabolic homeostasis. More broadly, PUFAs influence membrane biophysical properties, including membrane fluidity and lipid raft organization, thereby modulating receptor localization and signal transduction. In addition, they serve as precursors for specialized pro-resolving lipid mediators, such as resolvins and protectins, which actively promote the resolution of inflammation and support neuronal integrity [104,105].

Importantly, FFAs also function as direct nutrient signals within central homeostatic nuclei, particularly in the hypothalamus. The brain is capable of sensing circulating lipid availability and integrating this information to regulate feeding behavior, glucose metabolism, and energy expenditure. This process involves both passive uptake and protein-mediated transport across the blood–brain barrier, followed by intracellular metabolic and signaling events within neurons and astrocytes. Among the molecular mediators of lipid sensing, free fatty acid receptors (FFARs) have emerged as key components linking extracellular lipid availability to intracellular signaling pathways.

In particular, FFAR1/GPR40 has been identified as a relevant hypothalamic lipid sensor responsive to medium- and long-chain fatty acids. Although initially characterized in pancreatic β-cells, where it modulates insulin secretion, accumulating evidence indicates that FFAR1 is also expressed in the CNS and contributes to the regulation of neuronal activity and metabolic homeostasis. Genetic deletion of FFAR1 in POMC neurons results in hyperphagia, increased body weight, and impaired glucose metabolism, supporting a receptor-mediated role for long-chain fatty acids in CNS regulation of systemic energy balance [106,107]. In parallel, FFAR4/GPR120 functions as a sensor for unsaturated fatty acids and mediates many of their beneficial metabolic effects, including anti-inflammatory signaling and improved insulin sensitivity, further highlighting the importance of receptor-specific lipid signaling in the adipose tissue–CNS axis. Beyond receptor-mediated mechanisms, FFAs can also influence central signaling through receptor-independent pathways, including their incorporation into membrane phospholipids, modulation of lipid raft dynamics, and conversion into secondary lipid mediators such as ceramides, diacylglycerols, and eicosanoids. These processes amplify and diversify lipid signaling, linking peripheral lipid overload to intracellular stress responses and neuroinflammation.

Collectively, these findings indicate that the biological effects of fatty acids depend not simply on lipid availability but on their molecular characteristics and receptor specificity, which ultimately determine whether adipose tissue–CNS communication promotes metabolic adaptation or chronic inflammation.

#### 5.1.2. Ceramides and Lipotoxic Signaling

In addition to free fatty acids, lipid-derived intermediates such as ceramides, diacylglycerols, and eicosanoids contribute to intracellular and inter-organ signaling. Ceramides are especially relevant in obesity because excessive hypothalamic de novo ceramide synthesis has been linked to central insulin resistance, endoplasmic reticulum stress, inflammation, and dysregulation of glucose homeostasis. Thus, ceramides are increasingly viewed as lipotoxic mediators at the interface between nutrient excess and neuronal dysfunction [108,109]. Ceramides are generated through three main pathways: (i) de novo synthesis from serine and palmitoyl-CoA via serine palmitoyltransferase (SPT), (ii) sphingomyelin hydrolysis mediated by sphingomyelinases, and (iii) the salvage pathway, involving the recycling of sphingosine. Importantly, conditions of nutrient overload—particularly high levels of saturated fatty acids such as palmitate—strongly stimulate de novo ceramide synthesis, leading to their accumulation in metabolically active tissues, including adipose tissue, liver, and the central nervous system [110]. In adipose tissue, ceramides contribute to insulin resistance by interfering with canonical insulin signaling pathways. At the cellular level, ceramides act as potent second messengers that interfere with insulin signaling through multiple converging mechanisms. Mechanistically, ceramides inhibit Akt/protein kinase B activation through multiple routes, including activation of protein phosphatase 2A (PP2A) and atypical protein kinase C isoforms (PKCζ), ultimately impairing glucose uptake and promoting lipolysis dysregulation [108]. In parallel, ceramides promote mitochondrial dysfunction by altering membrane integrity and respiratory efficiency, and they induce endoplasmic reticulum stress and oxidative stress responses, thereby amplifying inflammatory signaling cascades such as JNK and NF-κB pathways [8,111]. These processes collectively contribute to the establishment of insulin resistance and metabolic inflexibility.

Within adipose tissue, ceramides exert pleiotropic effects on adipocyte biology and tissue remodeling. Their accumulation promotes adipocyte hypertrophy, impairs adipogenesis, reduces mitochondrial oxidative capacity, and enhances the production and secretion of pro-inflammatory cytokines, thereby fostering the chronic low-grade inflammatory state characteristic of obesity [8,112]. In addition, ceramides impair the function of thermogenic adipocytes by reducing mitochondrial respiration and β-adrenergic responsiveness, thereby decreasing energy expenditure and promoting weight gain [113]. These alterations further exacerbate systemic metabolic dysfunction and reinforce lipid spillover into non-adipose tissues.

Importantly, ceramides also participate in inter-organ communication, contributing to the adipose tissue–CNS axis. Circulating ceramides derived from peripheral tissues have been proposed to act as endocrine-like signals capable of influencing brain function, including the induction of central insulin resistance and neuroinflammatory responses [114]. In parallel, local ceramide accumulation within the hypothalamus plays a direct role in the dysregulation of energy balance. Excessive de novo ceramide synthesis in hypothalamic neurons induces endoplasmic reticulum stress, mitochondrial dysfunction, and impaired leptin and insulin signaling, ultimately disrupting neuronal circuits that control food intake, thermogenesis, and glucose homeostasis [108]. Recent experimental studies further demonstrate that specific ceramide species, such as those generated by ceramide synthase 6 (CerS6), promote hypothalamic lipotoxicity by inducing ER–mitochondrial stress and altering neuronal function, thereby contributing to obesity-associated metabolic impairment [115].

Overall, ceramides represent a key mechanistic link between peripheral nutrient overload and central metabolic dysfunction, acting as both intracellular effectors and mediators of adipose tissue–brain communication.

#### 5.1.3. DAGs, Oxylipins and S1

Beyond free fatty acids and ceramides, additional lipid-derived intermediates contribute to the regulation of metabolic and inflammatory signaling along the adipose tissue–CNS axis. Diacylglycerols (DAGs), eicosanoids/oxylipins, and sphingolipid metabolites such as sphingosine-1-phosphate (S1P) act as key signaling molecules linking nutrient overload to intracellular stress responses, inflammatory pathways, and inter-organ metabolic communication.

Diacylglycerols (DAGs) are glycerol-based lipid intermediates generated during triacylglycerol hydrolysis and phospholipid turnover. In conditions of chronic nutrient oversupply, DAGs accumulate in metabolically active tissues, including liver, skeletal muscle, and adipose tissue, where they act as second messengers capable of activating conventional and novel isoforms of protein kinase C (PKC). This activation interferes with insulin receptor signaling by promoting inhibitory phosphorylation of insulin receptor substrates, thereby contributing to insulin resistance [116,117]. Although the role of DAGs has been extensively characterized in peripheral tissues, emerging evidence suggests that similar mechanisms may also operate at the central level. In the hypothalamus, lipid-induced PKC activation has been associated with impaired insulin and leptin signaling, suggesting that DAG accumulation may participate in the development of central metabolic resistance and altered energy balance control [116]. Thus, DAGs can be viewed as intracellular transducers that amplify the effects of lipid overload within both adipose tissue and the CNS. Another major class of bioactive lipid mediators is represented by eicosanoids and related oxylipins, which are oxygenated derivatives of polyunsaturated fatty acids generated through enzymatic pathways involving cyclooxygenases (COX), lipoxygenases (LOX), and cytochrome P450 enzymes. These lipid mediators include prostaglandins, leukotrienes, thromboxanes, and specialized pro-resolving mediators such as resolvins and protectins. In obesity, the balance between pro-inflammatory and pro-resolving lipid mediators is profoundly altered, with increased production of pro-inflammatory eicosanoids derived from omega-6 fatty acids and a relative deficiency of anti-inflammatory and pro-resolving species [8,118]. This shift contributes to the maintenance of chronic low-grade inflammation in adipose tissue and promotes systemic metabolic dysfunction. At the CNS level, eicosanoids modulate neuroinflammatory processes by influencing microglial activation, cytokine production, and blood–brain barrier permeability. Prostaglandins, in particular, have been shown to affect hypothalamic neuronal activity and energy balance regulation, while specialized pro-resolving mediators derived from omega-3 fatty acids can counteract neuroinflammation and support neuronal function [118]. Therefore, oxylipins represent a critical interface between dietary lipid composition, inflammatory signaling, and central metabolic control. Within the sphingolipid pathway, sphingosine-1-phosphate (S1P) emerges as a functionally distinct metabolite that often exerts effects opposing those of ceramides. Generated through phosphorylation of sphingosine by sphingosine kinases, S1P acts both as an intracellular second messenger and as an extracellular ligand for a family of G protein–coupled receptors (S1PR1–S1PR5). This dual mode of action allows S1P to regulate a wide range of biological processes, including cell survival, inflammation, and vascular integrity [8,119]. In contrast to the pro-apoptotic and insulin-desensitizing effects of ceramides, S1P is generally associated with pro-survival and anti-inflammatory signaling, contributing to the maintenance of metabolic homeostasis. In the context of obesity, dysregulation of the ceramide/S1P rheostat—the dynamic balance between ceramide and S1P levels—has been proposed as a key determinant of cellular fate and metabolic function [8]. Within the CNS, S1P signaling influences neuroinflammatory responses, glial activity, and neuronal survival, and may therefore play a modulatory role in the central adaptations to metabolic stress [119].

Taken together, these lipid mediators illustrate how different classes of bioactive lipids exert complementary and sometimes opposing actions within the adipose tissue–CNS axis, emphasizing the complexity of lipid signaling beyond simple energy storage.

### 5.2. Nutrient-Sensing Receptors and Intracellular Signaling Pathways

Nutrients and their derived metabolites exert their biological effects not only through their metabolic utilization but also via direct interactions with specific cellular receptors, acting as signaling molecules that influence gene expression, neuronal activity, and systemic metabolism. These interactions involve multiple classes of receptors, including G protein–coupled receptors (GPCRs), nuclear receptors, and intracellular signaling hubs, which are widely expressed in both peripheral metabolic tissues and the central nervous system.

Among the membrane nutrient sensors, G protein–coupled receptors (GPCRs) represent a key interface between extracellular metabolites and intracellular signaling cascades. Free fatty acid receptors (FFARs) are particularly relevant in this context. FFAR1 (GPR40) and FFAR4 (GPR120) respond to medium- and long-chain fatty acids, modulating insulin secretion, inflammation, and neuronal activity. Notably, GPR120 has been identified as a lipid sensor with anti-inflammatory functions, and its dysfunction has been linked to obesity and metabolic disorders [120]. Moreover, activation of GPR120 in enteroendocrine cells stimulates incretin secretion, including GLP-1, thereby contributing to systemic metabolic regulation [121].

Short-chain fatty acids (SCFAs), such as acetate, propionate, and butyrate, also act as signaling molecules through GPCRs, primarily FFAR2 (GPR43) and FFAR3 (GPR41). These receptors are expressed in adipose tissue, immune cells, and the gut, where they regulate hormone secretion, inflammation, and energy homeostasis [122,123]. In addition, SCFA signaling through GPR43 has been shown to modulate insulin sensitivity and fat accumulation, highlighting a critical role of gut-derived metabolites in metabolic control [124]. At the central level, GPCR-mediated nutrient sensing contributes to the regulation of hypothalamic function and neuroinflammatory tone. Excess lipid exposure activates inflammatory pathways in hypothalamic microglia and neurons, impairing metabolic signaling [125]. Conversely, activation of lipid-sensing receptors such as GPR120 may counteract these processes by attenuating inflammatory signaling and preserving neuronal responsiveness to metabolic hormones.

In parallel, several nutrient-derived molecules act as ligands for nuclear receptors, which regulate transcriptional programs involved in lipid metabolism, inflammation, and metabolic adaptation. Peroxisome proliferator-activated receptors (PPARs) are central mediators of lipid signaling. PPARγ plays a major role in adipocyte differentiation and insulin sensitivity and also exerts anti-inflammatory effects [126]. More broadly, the PPAR family coordinates lipid metabolism, energy balance, and inflammatory responses across tissues. In the central nervous system, PPARs are involved in the modulation of neuroinflammation and neuronal metabolism, suggesting a role in the integration of peripheral metabolic signals with central regulatory pathways [127]. Other nuclear receptors, such as liver X receptors (LXRs), respond to sterol-derived ligands and regulate cholesterol homeostasis and inflammatory signaling [128]. Together, these receptor systems link nutrient-derived ligands to transcriptional programs that influence both adipose tissue function and CNS activity. Nutrients also modulate intracellular signaling pathways involved in cellular energy sensing. Amino acids, particularly leucine, activate the mechanistic target of rapamycin complex 1 (mTORC1), which integrates nutrient availability with anabolic processes and cellular growth. In the hypothalamus, mTOR signaling has been shown to regulate food intake and energy balance [129]. Conversely, AMP-activated protein kinase (AMPK) acts as a cellular energy sensor activated during low energy states. In the hypothalamus, AMPK integrates hormonal and nutrient signals to modulate feeding behavior and energy expenditure [130]. The dynamic balance between AMPK and mTOR signaling represents a critical node in central metabolic regulation [131,132]. In parallel, nutrient excess—particularly lipid overload—activates stress-related signaling pathways such as JNK and NF-κB, linking metabolic imbalance to inflammation and insulin resistance, as discussed in the previous sections.

### 5.3. Gut Microbiota-Derived Metabolites and Polyphenols as Modulators of the Adipose Tissue–CNS Axis

Beyond fatty acids and lipid-derived mediators, gut microbiota-derived metabolites represent an additional class of bioactive signals involved in adipose tissue–CNS communication. SCFAs, mainly acetate, propionate, and butyrate, are generated through microbial fermentation of dietary fibers and act as metabolic and immunomodulatory molecules. SCFAs signal through G protein-coupled receptors, including FFAR2/GPR43 and FFAR3/GPR41, which are expressed in intestinal enteroendocrine cells, adipose tissue, immune cells, and neural-related tissues. Through these receptors, SCFAs can promote the secretion of gut hormones such as glucagon-like peptide-1 (GLP-1) and peptide YY (PYY), thereby influencing satiety, glucose homeostasis, insulin sensitivity, and energy balance. In addition, SCFAs may modulate neuroinflammatory tone and gut–brain communication through vagal signaling, immune regulation, and blood–brain barrier integrity. In this perspective, SCFAs represent a mechanistic link between dietary fiber intake, gut microbiota activity, adipose tissue function, and central metabolic regulation [133,134]. Bile acids also act as microbiota-modified endocrine mediators involved in metabolic regulation. Primary bile acids synthesized in the liver are converted by gut microbiota into secondary bile acids, which activate receptors such as farnesoid X receptor (FXR) and Takeda G protein-coupled receptor 5 (TGR5). These receptors regulate lipid and glucose metabolism, GLP-1 secretion, energy expenditure, and inflammatory responses. TGR5 activation has been linked to enhanced thermogenesis and improved metabolic homeostasis, whereas FXR signaling contributes to bile acid, lipid, and glucose regulation. Therefore, bile acid signaling provides another molecular route through which diet, gut microbiota, adipose tissue metabolism, and CNS-related energy regulation may converge [135].

Polyphenols represent a further class of multifunctional bioactive compounds potentially able to modulate the adipose tissue–CNS axis. Beyond their classical antioxidant activity, polyphenols such as resveratrol, quercetin, epigallocatechin gallate, anthocyanins, and curcumin influence key obesity-related pathways, including AMPK, SIRT1, Nrf2, NF-κB, and PPAR signaling. Through these mechanisms, they may reduce adipose tissue inflammation, improve insulin sensitivity, attenuate oxidative stress, and limit hypothalamic neuroinflammation. Importantly, due to their limited intestinal absorption, many dietary polyphenols reach the colon, where they are transformed by the gut microbiota into smaller phenolic metabolites with systemic activity. This bidirectional interaction between polyphenols and gut microbiota may contribute to the modulation of gut barrier function, microbial composition, SCFA production, and inflammatory signaling, thereby reinforcing their role as pleiotropic modulators of the gut–adipose tissue–brain axis [136,137]. Taken together, SCFAs, bile acids, and polyphenol-derived metabolites expand the concept of nutrient signaling beyond classical macronutrient metabolism. These compounds act as molecular intermediates linking diet quality, microbial metabolism, adipose tissue inflammation, endocrine signaling, and CNS regulation of appetite and energy homeostasis. Their integration into the adipose tissue–brain axis supports the rationale for microbiota-targeted and bioactive compound-based nutritional strategies in obesity.

## 6. Metabolic Integration and Signaling Functions of White Adipose Tissue

WAT can be considered a central node in the metabolic and systemic communication networks of the organism. The integration of lipid metabolism, carbohydrate metabolism, and mitochondrial function not only determines the bioenergetic efficiency of the adipocyte but also represents the functional basis of its ability to communicate with other cell types and with distal organs. Indeed, through the release of extracellular vesicles, adipokines, lipid metabolites, and neural signals, WAT contributes to the coordinated regulation of energy homeostasis and to signal transmission between adipose tissue, the immune system, and the central nervous system.

### 6.1. Lipid and Carbohydrate Metabolism in Adipose Tissue

WAT is not a simple energy store, but a metabolically active organ that integrates lipid and carbohydrate metabolism to maintain systemic homeostasis [138]. Most of the fatty acids stored in WAT are derived from the hydrolysis of circulating triacylglycerols (TAGs) contained in VLDL and chylomicrons. After the action of lipoprotein lipase, fatty acids enter adipocytes by passive diffusion or via transporters such as CD36 and fatty acid-binding proteins [139,140]. In the adipocyte, fatty acids are then converted to acyl-CoA and esterified to glycerol-3-phosphate to form TAG, by enzymes such as GPAT, AGPAT, lipins, and DGAT [141,142].

Lipid mobilization is activated during fasting or exercise and is hormonally regulated, primarily by insulin and other peptides. TAG degradation is mediated by three major neutral lipases: adipose triglyceride lipase (ATGL, encoded by PNPLA2), responsible for the hydrolysis of TAGs to diacylglycerols (DAGs); hormone-sensitive lipase (HSL), which hydrolyzes DAGs and partly TAGs; and monoglyceride lipase, which completes the degradation of monoacylglycerols to glycerol and fatty acids [143,144,145]. Their activity is modulated by their interaction with lipid droplet proteins. An example is Perilipin 1 (PLIN1), which, once phosphorylated, interacts with ATGL and also stimulates HSL activity [146]. Alongside classical lipolysis, alternative lipid mobilization pathways such as lipophagy, a form of macroautophagy stimulated by β-adrenergic receptors, also contribute to TAG degradation [147].

Carbohydrate metabolism is regulated by insulin, which stimulates the translocation of glucose transporters to the adipocyte membrane [148]. Glucose is not only used for energy production, but also fuels de novo lipogenesis (DNL), a process associated with insulin sensitivity and regulated by ChREBP [149,150]. De novo fatty acid synthesis closely links carbohydrate and lipid metabolism. Specifically, glucose enters the adipocyte via insulin-sensitive (GLUT4) and non-sensitive (GLUT1) transporters, and is metabolized through glycolysis and the TCA cycle to produce citrate. ACLY and ACC1 generate acetyl-CoA and malonyl-CoA, respectively, which are used by FASN to synthesize palmitic acid [151]. Furthermore, there is functional heterogeneity among white adipocytes, with subpopulations characterized by a prevalent glycolytic or lipogenic activity [152,153]. Glucose metabolism is also intertwined with that of branched-chain amino acids (BCAA: leucine, isoleucine, and valine). Although BCAAs have beneficial effects on protein synthesis under conditions such as aging or cachexia, elevated plasma concentrations are associated with obesity, insulin resistance, type 2 diabetes, and cardiovascular disease [154,155]. The increase in circulating BCAA levels is partly attributable to reduced oxidation in WAT, due to the suppression of catabolic enzymes [156,157].

### 6.2. Role of Mitochondria and Cellular Bioenergetics

The function of WAT is deeply linked to mitochondrial dynamics. During adipogenesis, a reorganization of lipid and oxidative metabolism is required, mediated by mitochondrial signals and signaling pathways such as PPARγ/PGC1α, WNT, and NOTCH2 [158,159,160]. In fact, correct mitochondrial biogenesis supports lipolysis and fatty acid oxidation; on the contrary, mitochondrial dysfunctions compromise lipid and carbohydrate metabolism [161].

In recent years, it has emerged that mitochondria in white adipose tissue not only play a bioenergetic role, but also act as key regulators of progenitor cell differentiation fate and mature adipocyte function. The identification of mitochondrial regulators and miRNAs opens new perspectives for improving adipose health [162].

Mitochondrial metabolism plays a key role in the differentiation of adipose progenitor cells (APCs). Furthermore, mitochondrial dysfunction in APCs leads to increased adipose tissue inflammation and partial lipodystrophy [163].

Some studies in mouse models have analyzed the role of mitochondrial regulation in adipocyte function. For instance, deletion of peroxiredoxin 3 (Prx3), a mitochondrial peroxidase whose presence is abundant in white adipocytes but which is reduced under obese conditions, is observed to interfere with mitochondrial biogenesis, and is associated with increased oxidative stress and insulin resistance [164]. Similarly, removal of Paraoxonase 2 (PON2), a mitochondria-localized lactonase, reduces mitochondrial respiration and causes adipocyte hypertrophy in WAT, thereby promoting diet-induced obesity [165]. Finally, deletion of the mitochondrial dicarboxylate transporter (mDIC) has also been associated with lipotoxicity as it leads to excessive release of fatty acids. mDIC is highly expressed in WAT and controls the export of succinate from mitochondria to the cytosol and consequently lipolysis [166].

Mitochondrial DNA in adipocytes encodes proteins essential for respiration and cellular energy, yet it is vulnerable to oxidative damage and epigenetic modifications that can impair adipocyte metabolism and promote obesity, hepatic steatosis, and insulin resistance. mtDNA repair and its proper maintenance, regulated by enzymes such as NEIL1 and OGG1 and by adipogenesis, are critical for the metabolic function and bioenergetics of adipocytes [167,168,169,170].

Mitochondrial fusion and fission dynamics are critical for mitochondrial homeostasis. Outer membrane fusion is mediated by MFN1 and MFN2, the latter also involved in mitochondrion-lipid droplet and mitochondrion-ER interactions, while inner membrane fusion is regulated by OPA1, modulated by OMA1 [171,172,173,174,175,176]. Fission is controlled by DRP1, FIS1, MFF, MiD49, and MiD51 [177,178]. Dysfunctions in MFN2 or OPA1 impair oxidative phosphorylation, glucose and fatty acid oxidation, and membrane potential, while alterations in DRP1 and FIS1 affect triglyceride accumulation [179,180].

Mitophagy represents an additional quality control mechanism, used by the cell to specifically eliminate damaged mitochondria. The specific receptor FUNDC1 is essential for this process in adipocytes and its deficiency compromises mitophagy and increases the inflammatory state in adipose tissue. Furthermore, loss of FUNDC1 function is associated with worsening diet-induced obesity [181].

### 6.3. White Adipose Tissue as a Signaling Hub

WAT emerges as an endocrine and neuro-metabolic organ actively involved in intercellular crosstalk networks and in the bidirectional modulation of the brain–adipose axis. Indeed, mitochondria in WAT and at the systemic level do not exclusively regulate energy homeostasis; rather, they actively participate in intercellular communication both within adipose tissue and toward distal organs [162]. Mitochondria can be transferred intercellularly, primarily from stem cells to other cell types; this mechanism contributes to metabolic and energetic synchronization between distinct cellular populations [182]. In WAT, this transfer occurs from adipocytes to macrophages through heparan sulfate–mediated mechanisms; under obese conditions, this process is reduced and is associated with increased adiposity, decreased energy expenditure, and impaired glucose tolerance in murine models [183].

Beyond direct organelle transfer, WAT releases a substantial number of extracellular vesicles (EVs) in response to metabolic stimuli, which may contain functional mitochondria or mitochondrial components [184,185,186,187,188]. EVs carrying mitochondrial material are capable of modifying the energetic state of recipient cells; notably, in the heart, mitochondrial vesicles derived from metabolically compromised adipocytes have been shown to induce moderate ROS production, thereby preconditioning the myocardium to more severe oxidative stress [188].

Within WAT, EVs are secreted by multiple cell types, including adipocytes, macrophages, adipose-derived stem cells (ADSCs), and endothelial cells, forming a local and systemic communication network that contributes to metabolic homeostasis [189]. The amount of EVs produced by adipocytes varies according to metabolic status and hormonal stimulation; they contain adiponectin and other adipocyte-specific proteins such as perilipin A and FABP4 [190,191,192,193,194]. Adipocyte-derived EVs modulate macrophage activity. In vitro, adiponectin-positive EVs promote monocyte differentiation into adipose tissue macrophages (ATMs), inducing the expression of both pro- and anti-inflammatory markers [192]. Conversely, ATMs also secrete EVs: exosomes derived from M1 macrophages inhibit insulin signaling in adipocytes, whereas those from M2 macrophages enhance it, highlighting the role of macrophages in metabolic homeostasis and inflammation [195]. In vivo, infusion of exosomes from lean ATMs improves glucose intolerance and insulin resistance in obese mice, whereas exosomes from obese ATMs worsen these parameters in lean mice [196].

Adipose-derived stem cells further contribute to paracrine communication through EV release. Administration of stem cell–derived exosomes in obese mice attenuates obesity and insulin resistance by promoting macrophage polarization toward the M2 phenotype via STAT3-mediated transactivation of arginase-1, thereby favoring WAT beginning [197]. The EV cargo of adipose tissue includes miRNAs, mRNAs, lncRNAs, proteins, and lipids [189].

miRNAs contained in adipocyte- and macrophage-derived EVs—such as miR-34a, miR-155, and miR-27a—regulate macrophage polarization, insulin signaling, and lipid metabolism in distal tissues [196,198,199,200,201]. Adipocyte-derived EVs also transport metabolic mRNAs and regulatory lncRNAs, including lnc-BATE1 and lnc-MALAT1, which modulate adipocyte differentiation and hypothalamic circuits controlling appetite and thermogenesis [202,203,204,205]. Exosomal proteins such as RBP4 and STAT3 influence macrophage polarization toward pro- or anti-inflammatory phenotypes, while lipid-rich exosomes transfer lipids and α-ketoglutarate to macrophages, promoting M2 responses and reducing adipose inflammation [191,197,206].

Beyond peripheral effects, adipose tissue–derived EVs also participate in communication with the central nervous system. Adipose-derived EVs have been shown to cross the blood–brain barrier and reach the brain [207]. In the context of diabetes, these EVs may induce synaptic loss and cognitive decline; in both diabetic patients and murine models, vesicle-carried miRNAs reach the hippocampus, inducing synaptic damage and cognitive impairment [207]. A potential role in this process has been attributed to miR-9-3p, which is upregulated in the hippocampus and in adipocyte-derived EVs in both diabetic mice and patients [207]. Elevated levels of the same miRNA are associated with memory deficits in Alzheimer’s disease models [208,209]. These findings delineate an EV-mediated adipose–brain axis that, under conditions of metabolic dysfunction, may contribute to synaptic damage and cognitive decline [207].

In addition to EV-mediated communication, WAT–brain interactions are regulated by well-defined neuroanatomical circuits. WAT is innervated by the autonomic nervous system. Different adipose depots (e.g., subcutaneous and visceral) display distinct innervation density, receptor expression profiles, and differential sympathetic activity [14,210]. At the sympathetic level, norepinephrine (NE) acts on β-adrenergic receptors (βARs), enhancing lipolysis and the release of free fatty acids (FFAs) and glycerol from white adipocytes [10]. The role of the parasympathetic system remains debated; however, parasympathetic innervation of WAT has been implicated in leptin and resistin production and in the regulation of insulin sensitivity [211].

The central nervous system exerts its effects on WAT primarily via the sympathetic system, whose activity is modulated by POMC and AgRP neurons in response to nutritional status [32,33]. Leptin exerts “top-down” control over WAT sympathetic innervation through ARC–PVH circuits dependent on BDNF [34,212], whereas hypothalamic insulin reduces sympathetic activity and promotes lipogenesis [213]. Conversely, WAT sends afferent signals to the brain. Communication occurs through pseudo-unipolar neurons located in the dorsal root ganglia (DRG) of the spinal cord [214]. Through various receptors, these neurons detect the metabolic state of WAT and transmit information necessary for the modulation of whole-body lipid balance [215].

Adipose tissue secretes multiple molecules that act centrally by modulating brain regions involved in energy homeostasis. Among these are adipokines, the principal one being leptin. Leptin acts on the arcuate nucleus by activating POMC neurons and inhibiting AgRP neurons, thereby regulating energy expenditure, insulin sensitivity, and satiety [216,217,218]. Adiponectin, via hypothalamic AdipoR1/R2, influences food intake, energy metabolism, glucose homeostasis, and circadian rhythms, also exerting effects on astrocytes and oxytocinergic neurons [219,220,221,222]. Resistin regulates sympathetic activity and interacts with leptin at the hypothalamic level [223,224]. These findings underscore the role of adipokines in bidirectional communication between WAT and the brain and in the integrated regulation of energy balance [224].

WAT also secretes fatty acids that circulate as triglycerides or albumin-bound species and, via protein transporters, can cross the blood–brain barrier [225,226,227]. Once in the brain, fatty acids may be incorporated into membrane phospholipids or act as signaling molecules, regulating hypothalamic neurons such as AgRP and POMC cells, thereby influencing food intake, glucose production, insulin action, and hepatic lipoprotein secretion [107,228,229,230,231,232,233].

Beyond neural, hormonal, and lipid-mediated signaling, recent evidence has identified additional layers of complexity in adipose–brain communication. Neuro-mesenchymal units represent cellular networks in which adipose mesenchymal cells and sympathetic neurons functionally cooperate. These units regulate innate immune cells such as ILC2s via β2-adrenergic receptors; their dysfunction alters cytokine production and contributes to metabolic imbalance [11,234].

### 6.4. Temporal Sequence and Spatial Heterogeneity in Adipose–CNS Crosstalk

Addressing the temporal sequence of events is crucial: is CNS dysfunction a cause or a consequence of adipose tissue expansion? Preclinical data suggest that hypothalamic neuroinflammation is a remarkably early event, occurring within 24 to 48 h of high-fat diet (HFD) exposure in rodents, long before any measurable increase in body weight or adipose tissue mass occurs. This observation suggests that acute nutritional lipid overload may directly affect the CNS and contribute to the initiation of hypothalamic dysfunction. Such early central alterations may disrupt sympathetic output, reducing energy expenditure and altering peripheral lipid metabolism, thereby favoring adipose tissue expansion. Once adipose tissue expands, chronic low-grade inflammation and altered adipokine secretion feed back to the brain, further reinforcing metabolic dysfunction. Although experimental evidence supports an early hypothalamic response to nutritional excess, the relative contribution of primary CNS dysfunction versus adipose-derived inflammatory signaling in human obesity remains incompletely resolved.

This bidirectional signaling is strongly influenced by spatial heterogeneity. Visceral adipose tissue (VAT) and subcutaneous adipose tissue (SAT) exhibit markedly different impacts on the brain. VAT is drained by the portal circulation and is particularly susceptible to hypoxia, immune-cell infiltration, and inflammatory remodeling. Consequently, VAT releases higher levels of pro-inflammatory cytokines (e.g., IL-6 and TNF-α), ceramides, and other lipid mediators that may contribute to blood–brain barrier dysfunction, hypothalamic inflammation, and central insulin resistance. In contrast, SAT has a greater capacity for healthy expansion, is more prone to β3-adrenergic-induced browning, and generally exhibits a more favorable secretory profile characterized by higher adiponectin production and lower inflammatory activity. These features may promote a more neuroprotective and metabolically resilient environment. Therefore, the anatomical origin and biological characteristics of adipose depots represent important determinants of the quality and physiological impact of peripheral signals reaching the CNS.

## 7. Impact of Dietary Patterns on the CNS–Adipose Tissue Axis

Adipose tissue and the central nervous system (CNS) form a bidirectional regulatory axis that integrates metabolic, immune and neuroendocrine signals. Adipose tissue acts as an endocrine organ, releasing adipokines and cytokines that inform the CNS about energetic status, while the CNS modulates adipose tissue function and distribution through sympathetic pathways and hypothalamic circuits [235].

Dietary patterns rich in saturated fats and refined sugars promote obesity, insulin resistance and chronic low-grade inflammation, all conditions associated with microglial activation, neuroinflammation and increased risk of neurological and psychiatric disease [236,237]. Peripheral inflammatory signals and adipokines modulate microglial reactivity, altering synaptic plasticity and behavioral outputs [238].

Dysregulated diets also remodel the microbiota–gut–brain axis, promoting dysbiosis, vagal and cytokine signaling alterations, and hypothalamic inflammation, with consequent disruption of homeostatic energy-control circuits [239,240]. From a functional standpoint, diet-induced obesity is increasingly interpreted not as a failure of central regulatory circuits but as an upward shift in the defended adiposity level, which makes weight loss resistant in obesogenic environments [241]. Additionally, high-fat feeding reduces insulin transport to the CNS, impairing negative feedback mechanisms regulating adiposity [242].

Dietary lipids directly influence the CNS by crossing the blood–brain barrier or by shaping local lipid metabolism. Alterations in brain lipid homeostasis contribute to Alzheimer’s disease, Parkinson’s disease and stroke pathogenesis [243]. Dysfunctions of the microbiota–gut–brain axis also involve adipose tissue as a dynamic immunometabolic organ interacting with microbial metabolites and immune cells, amplifying systemic metabolic stress [244]. Microbial products such as short-chain fatty acids and tryptophan-derived metabolites modulate neuroinflammation, glial activation and amyloid deposition, establishing mechanistic links between diet, microbiota and neurodegeneration [245].

Conversely, dietary patterns such as the Mediterranean and MIND diets—rich in polyphenols, omega-3 fatty acids and antioxidant compounds—are consistently associated with better cognitive trajectories and reduced risk of neurodegenerative decline [246]. Nutrients with antioxidant and anti-inflammatory properties modulate oxidative stress, neurotransmission and epigenetic processes, contributing to lower incidence of depression and anxiety [247].

In conclusion, mechanistic evidence shows that dietary patterns exert profound effects on the CNS–adipose tissue axis by modulating inflammatory, neuroendocrine and metabolic pathways. Western dietary patterns induce maladaptive remodeling of central energy-control circuits and microglial function, whereas nutrient-dense dietary models enhance neuronal resilience and metabolic homeostasis. Nevertheless, most evidence derives from preclinical and review studies; therefore, large-scale controlled trials are required to quantify clinical outcomes with higher precision. Table 1 provides an integrated summary of key studies on dietary modulation of the CNS–adipose tissue axis.

### 7.1. Diet, Inflammation and Appetite Regulation

Diet is a key modulator of both systemic inflammation and appetite regulation, two interconnected processes underlying obesity. Evidence from randomized controlled trials indicates that dietary composition can influence inflammatory status independently of weight loss. For example, almond consumption significantly reduced circulating pro-inflammatory cytokines such as IL-6, TNF-α and IFN-γ without affecting body weight or appetite [248]. Similarly, specific dietary manipulations, such as carbohydrate distribution, were associated with reductions in inflammatory markers (CRP, IL-6, TNF-α) alongside improvements in leptin and adiponectin profiles [249]. However, other interventions, including variation in eating frequency, showed no significant effects on inflammatory biomarkers or appetite-related hormones, highlighting heterogeneity in responses [250].

Diet also directly modulates appetite regulation through endocrine and metabolic pathways. Wholegrain-based interventions have been shown to influence postprandial ghrelin and glycemic control, although effects on incretins are inconsistent [251]. Likewise, manipulation of meal timing significantly alters hunger and appetite-regulating hormones, with late eating increasing hunger and the ghrelin/leptin ratio, thereby promoting a positive energy balance [252]. Other dietary strategies, including low-glycemic index diets, prebiotic fibers, and resistant carbohydrates, have demonstrated variable effects on satiety, inflammatory markers, and metabolic outcomes [253,254,255]. Acute dietary factors such as advanced glycation end products may also modulate appetite-related hormones like ghrelin, although effects on inflammation appear limited [256].

Mechanistically, increasing evidence supports a role for diet-induced inflammation in disrupting central appetite regulation along the adipose tissue–central nervous system axis. A systematic review in International Journal of Molecular Sciences highlights that high-fat diets, particularly those rich in saturated fatty acids, promote hypothalamic inflammation through ceramide accumulation, endoplasmic reticulum stress, and lipotoxicity, ultimately leading to leptin and insulin resistance and impaired energy balance control [257]. Complementary experimental evidence in Brain, Behavior, and Immunity demonstrates that dietary fatty acid composition, especially a high ω6/ω3 ratio, induces neuroinflammation in key brain regions such as the hypothalamus and hippocampus, contributing to altered feeding behavior and metabolic dysfunction [258].

Overall, current evidence indicates that diet influences both peripheral inflammation and appetite-regulating hormones, while mechanistic studies suggest that these effects converge at the level of the central nervous system. However, although RCTs consistently show that dietary interventions can modulate inflammatory markers and appetite signals, direct evidence linking these changes to hypothalamic dysfunction in humans remains limited, with most mechanistic insights derived from preclinical models. Table 2 summarizes evidence from RCTs and mechanistic studies showing that dietary composition influences both peripheral inflammation and central appetite regulation.

However, considerable inter-individual variability exists in the response to dietary interventions. Genetic background, baseline metabolic status, sex-related differences, gut microbiota composition, and environmental factors may all influence inflammatory responses, appetite regulation, and adipokine signaling. These variables likely contribute to the heterogeneous outcomes reported across nutritional studies and support the development of more personalized dietary approaches.

### 7.2. Nutritional Strategies for Improving Metabolic Health

Nutritional strategies targeting metabolic health increasingly emphasize whole dietary patterns, macronutrient quality, and gut microbiota modulation. Adherence to a Mediterranean dietary pattern has been shown to improve metabolic syndrome components and inflammatory status in adolescent girls with metabolic syndrome, including reductions in IL-6 and hs-CRP alongside improvements in insulin resistance and lipid profiles [259]. Similarly, controlled interventions comparing Mediterranean-like diets to Western patterns report enhanced postprandial glucose metabolism and insulin sensitivity, partly mediated by increased production of short-chain fatty acids (SCFAs) and favorable shifts in gut microbiota composition [260].

Beyond the Mediterranean model, other healthy dietary patterns such as the Nordic diet also exert anti-inflammatory effects at the molecular level, modulating gene expression pathways related to immune and metabolic regulation [261]. Likewise, traditional dietary patterns such as the Atlantic diet have shown beneficial effects on body weight and lipid profiles in population-based interventions, supporting the role of culturally adapted dietary strategies in metabolic risk reduction [262].

Dietary fiber intake represents a key mechanistic link between nutrition and metabolic health. Whole-grain interventions increase circulating SCFAs, particularly propionate, which are associated with improved insulin responses and metabolic regulation [263]. In line with this, diets rich in plant-based foods and fermentable substrates promote microbiota-derived metabolites that influence host metabolism and inflammation.

Macronutrient quality also plays a central role. Diets enriched in unsaturated fatty acids and fiber—such as those including avocado—have been associated with improvements in cardiometabolic risk markers and reductions in inflammatory biomarkers, even in the absence of weight loss [264]. Similarly, inclusion of specific nutrient-dense foods such as mango or almonds may contribute to improved insulin sensitivity or dietary quality, although evidence remains less consistent and often secondary to overall dietary patterns [265,266]. Overall, current evidence supports a model in which nutritional strategies improve metabolic health through integrated mechanisms involving inflammation reduction, modulation of adipose tissue function, and gut microbiota-derived signaling pathways. These findings are supported by evidence from randomized controlled trials (Table 3), highlighting potential mechanisms linking diet, inflammation, gut microbiota, and CNS–adipose tissue signaling.

### 7.3. Methodological Limitations of Experimental Models and Barriers to Clinical Translation

Most of the mechanistic evidence regarding bidirectional communication within the adipose tissue–CNS axis comes from preclinical animal models, particularly rodents subjected to high-calorie diets (HFDs) or genetic modifications (e.g., Nscl-2 mutant mice). Although these models provide fundamental insights into hypothalamic cell biology and signaling pathways, their direct clinical translation to the obese patient has substantial intrinsic limitations that require caution in generalizing the findings. One of the main methodological discrepancies lies in species-specific differences in the biology of immune mediators and adipokines. A paradigmatic example is resistin: in rodents, this molecule is synthesized and secreted predominantly by adipocytes in white adipose tissue, showing direct fluctuations based on sex and adipose depot. In contrast, in humans, resistin expression by mature adipocytes is extremely modest, while its primary source of production is macrophages infiltrating visceral adipose tissue. Therefore, the sympathetic overactivation and hepatic insulin resistance experimentally induced in mice by centrally administered resistin reflect an endocrine mechanism that in humans instead takes on a predominantly paracrine connotation and is linked to the intensity of local macrophage inflammation. Furthermore, mouse models of HFD-induced obesity often utilize acute dietary exposures with extreme lipid percentages, which do not faithfully mimic the structural complexity of the human diet (characterized by matrix effects, biochemical interactions of polyphenols, and industrial or thermal modifications of foods, such as the generation of AGEs and acrylamide). Although randomized controlled trials (RCTs) in humans have validated the ability of complex nutritional interventions (such as the Mediterranean diet, the Nordic diet, or the supplementation of functional foods such as almonds and avocado) to reduce peripheral inflammatory biomarkers (IL-6, TNF-α, hs-CRP) and improve systemic parameters such as the HOMA-IR index, direct evidence of resolution of hypothalamic neuroinflammation or modification of the brain metabolome in humans remains limited by ethical and technical barriers to obtaining direct tissue data from the CNS. Most human clinical outcomes must therefore rely on surrogate CSF biomarkers or structural and functional neuroimaging techniques. To bridge this gap, future research must plan large-scale controlled trials capable of quantifying clinical outcomes more precisely, leveraging technological innovations such as nanoencapsulation or 3D printing to standardize the actual bioaccessibility of therapeutic compounds.

Recent advances in the field of high-resolution molecular characterisation and functional neuroscience have significantly expanded our understanding of the adipose tissue–central nervous system axis. These technologies provide complementary information, ranging from cellular heterogeneity and spatial organisation to functional neuronal connectivity and circulating biomarkers. However, each approach has specific strengths and limitations that must be taken into account when interpreting experimental results and translating them into clinical applications (Table 4).

Rather than competing approaches, these technologies should be viewed as complementary tools whose integration is expected to accelerate the translation of mechanistic discoveries into clinically relevant biomarkers and therapeutic strategies.

## 8. Food Matrix, Nutrient Bioavailability and Food Processing in the Obesity Context

The effectiveness of conventional nutritional interventions is often constrained by the limited biological availability and delivery of dietary bioactive compounds. This translational gap has encouraged the development of innovative food technologies designed to improve their stability, absorption, and metabolic efficacy, thereby providing a rationale for their application to the adipose tissue–CNS axis.

### 8.1. The Food Matrix: Structure, Digestion, and Metabolic Signaling

Foods consist of a complex chemical composition that determines their physicochemical and technological properties, while also influencing digestibility, bioavailability, absorption kinetics, and downstream metabolic signaling. Beyond the mere chemical composition of individual food components, it is recognized that the three-dimensional structure of food (e.g., the integrity of the cell wall, protein–lipid–carbohydrate interactions, colloidal architecture, and gel networks) critically determines the rate and extent of nutrient release during gastrointestinal transit, modulating postprandial glycemic and lipemic profiles, the secretion of intestinal hormones, and the sensation of satiety [267,268]. At the structural level, the food matrix functions through several distinct but interconnected mechanisms. The physical encapsulation of nutrients within cellular structures represents the fundamental matrix effect. In plant-based foods, the integrity of cell walls composed of β-glucans, arabinoxylans, pectins, and cellulose creates a physical barrier that limits enzymatic access to intracellular starch granules and lipid bodies. This slows hydrolysis rates and reduces glycemic and insulinemic responses to carbohydrate intake [269,270]. Furthermore, slower nutrient absorption may prolong the stimulation of ileal L cells, which secrete glucagon-like peptide-1 (GLP-1) and peptide YY (PYY), thereby enhancing satiety signaling and reducing energy intake through gut–brain axis mechanisms [271,272].

Protein–carbohydrate and protein–lipid interactions within the food matrix are additional determinants of nutrient bioavailability. In dairy matrices, for example, the micellar structure of casein and the membrane organization of milk fat globules profoundly influence the rate of protein hydrolysis and lipid digestion [273]. Protein-matrix interactions also modulate the release of bioactive peptides during gastrointestinal digestion, since protease accessibility to cleavage sites depends largely on protein conformation and structural organization [274]. The colloidal and emulsifying properties of the food matrix are a key factor in the bioavailability of lipophilic bioactive compounds. Carotenoids, fat-soluble vitamins, polyphenols, and long-chain polyunsaturated fatty acids are incorporated into lipid phases such as oil droplets, lamellar liquid crystals, and lipid bodies enclosed within organelle membranes. Their transfer into mixed micelles during intestinal digestion depends on the efficiency of lipolysis, bile salts emulsification, and the structural properties of the surrounding matrix [275,276]. The presence of co-ingested lipids can significantly improve the bioavailability of lipophilic compounds by promoting micelle formation, while the physical state of the lipid phase modulates the rate of lipolysis and absorption [277,278]. The bioavailability of polyphenols is particularly sensitive to matrix effects because these compounds interact with food macromolecules. Polyphenols can form non-covalent complexes with proteins through hydrophobic interactions and hydrogen bonds, as well as covalent bonds with cell wall polysaccharides. These mechanisms may reduce their extractability and bioaccessibility in the upper gastrointestinal tract [279]. Interestingly, matrix-bound polyphenols may reach the colon relatively intact, where they can serve as substrates for microbial biotransformation into smaller phenolic metabolites, such as urolithins, equol, and hydroxycinnamic acid derivatives, which may exhibit greater bioavailability and systemic anti-inflammatory activity than the original compounds [280].

The rheological and textural properties of the food matrix, including viscosity, gelling strength, and particle size, also modulate gastrointestinal transit time, chyme viscosity, and the mechanical stimulation of intestinal mechanoreceptors, thereby contributing to appetite regulation and neuroendocrine signaling. Soluble viscous fibers, such as oat and psyllium β-glucan, increase luminal viscosity by reducing the rate of glucose absorption and attenuating postprandial insulin secretion, stimulating the release of satiety hormones through prolonged exposure of nutrients to L cells in the distal small intestine [281,282]. Reducing particle size through grinding, homogenization, or high-pressure treatment alters the cellular architecture, increasing starch and protein digestibility while reducing the functional properties of dietary fiber. This may shift the metabolic response toward a higher glycemic profile and lower satiety potential [283].

### 8.2. Food Processing Effects on Food Properties in the Obesity Context

Food processing encompasses a broad spectrum of physical, chemical, thermal, and biological operations applied to raw materials in order to transform them into food products and improve their safety, shelf life, palatability, and technological functionality. Although certain processing techniques may preserve or enhance the functional and nutritional properties of foods, others can disrupt their native structural organization, thereby modifying nutrient bioaccessibility, digestion kinetics, metabolic availability, and postprandial endocrine responses [284]. In this perspective, understanding the mechanistic consequences of specific processing operations is essential for designing food products that may support metabolic homeostasis and contribute to the prevention or management of obesity-related metabolic dysfunction. Mechanistically, these processing-induced changes can modulate the adipose tissue–CNS axis by influencing adipose tissue inflammation, adipokine and cytokine secretion, gut hormone responses, microbiota-derived metabolites, and circulating lipid profiles. In turn, these peripheral signals may affect hypothalamic nutrient sensing, insulin and leptin responsiveness, neuroinflammatory pathways, and autonomic regulation of adipose tissue. Therefore, the metabolic effects of food processing should be interpreted not only in terms of nutrient composition, but also according to how processing shapes the peripheral and central signals involved in adipose tissue–brain communication.

#### 8.2.1. Thermal Processing

The effects of heat treatment on food matrix integrity and the stability of bioactive compounds depend largely on the intensity and duration of heat exposure, the water activity of the system, and the chemical nature of the compounds involved. Heat treatment at moderate temperatures can improve protein digestibility by inducing partial denaturation and unfolding, which increases protease accessibility to cleavage sites and may enhance amino acid bioavailability [285]. At the same time, starch gelatinization during cooking disrupts the semi-crystalline structure of starch granules, increasing their susceptibility to amylase-mediated digestion and potentially raising the glycemic index of foods. However, once cooled after gelatinization, a portion of the starch undergoes retrogradation, forming a starchy structure that resists digestion in the small intestine and reaches the colon as a fermentable substrate for the production of short-chain fatty acids by the microbiota, thereby exerting beneficial effects on insulin sensitivity and gut–brain axis signaling [286,287]. Moderate heat treatment may also improve the bioavailability of certain phytochemicals. In tomatoes, for example, thermal processing promotes the isomerization of lycopene from the all-trans to the cis configuration, which is generally considered more bioavailable. In addition, the disruption of chloroplast membranes can release carotenoids from protein–pigment complexes, facilitating their incorporation into lipid micelles during digestion [288]. At higher temperatures and in the presence of reducing sugars and amino acids, the Maillard reaction generates various advanced glycation end products (AGEs), volatile aromatic compounds, and brown pigments. AGEs exert pro-inflammatory and pro-oxidant effects through interaction with their specific receptor RAGE (receptor for advanced glycation products) by activating NF-κB-dependent inflammatory cascades in peripheral tissues and the central nervous system [289]. At the same time, high-temperature processing can lead to the formation of acrylamide, which may interfere with dopaminergic neurotransmission and hypothalamic signaling at significant exposure levels [290].

#### 8.2.2. Mechanical Processing and Particle Size Reduction

Mechanical processes, including milling, crushing, homogenization, and high-pressure treatment, exert their primary effects through the physical disruption of the food’s microstructure, affecting both the kinetics of nutrient release and the functionality of the matrix. The removal of bran and germ fractions during milling for the production of refined flour eliminates most of the dietary fiber, vitamins, minerals, and phytochemicals concentrated in the outer layers of the grain, while the reduction in particle size of the remaining endosperm drastically increases the surface area of the starch and its susceptibility to amylolytic digestion [291]. The consumption of refined grains is associated with elevated postprandial blood glucose levels, reduced satiety, and an increased risk of type 2 diabetes and obesity effects that are directly attributable to the loss of the matrix’s integrity rather than to changes in its chemical composition [292].

High-pressure processing (HPP) induces the non-thermal inactivation of microorganisms and enzymes, while preserving heat-sensitive vitamins, polyphenols, and volatile aromatic compounds. HPP can modify protein conformation and starch structure in ways that may improve protein digestibility and increase resistant starch content, potentially contributing to improved glycemic and satiety profiles in processed foods [293]. In plant matrices, HPP may disrupt cell wall structures, increasing the extractability and bioaccessibility of intracellular phenolic compounds and carotenoids, with potential implications for antioxidant and anti-inflammatory activity [294]. Ultrasonic treatment is a non-thermal technology that generates acoustic cavitation, producing localized high-pressure and high-temperature events capable of breaking down cellular structures, reducing particle size, improving emulsification, and enhancing the efficiency of extracting bioactive compounds from plant matrices [295].

#### 8.2.3. Fermentation

Fermentation plays an important role in the adipose tissue–central nervous system axis because of its ability to simultaneously modify the structure of the food matrix, improve nutrient bioavailability, generate bioactive metabolites, and modulate the composition and activity of the gut microbiota. Microbial fermentation by lactic acid bacteria, bifidobacteria, yeasts, and molds employs a broad enzymatic profile that destroys antinutritional factors, modifies macromolecular structures, and releases bioactive compounds from their bound forms [296]. For example, phytate hydrolysis during the fermentation of grains and legumes significantly improves the bioavailability of minerals such as iron, zinc, calcium, and magnesium. Deficiencies or suboptimal status of these micronutrients may occur in individuals with obesity and have been associated with impaired mitochondrial function and dysregulation of insulin signaling [297].

The hydrolysis of casein during the fermentation of dairy products generates peptides such as β-casomorphins, which interact with opioid receptors in the gastrointestinal tract and potentially in the central nervous system, modulating intestinal motility and appetite regulation via vagal afferent pathways [298]. The fermentation of soy generates peptides that inhibit dipeptidyl peptidase-4 (DPP-4), the enzyme responsible for inactivating the incretin hormones GLP-1 and glucose-dependent insulinotropic polypeptide (GIP), thereby prolonging their appetite-suppressing and insulin-secreting effects and contributing to an improvement in postprandial glucose metabolism [299]. Fermentation also promotes the biotransformation of complex polyphenols, such as ellagitannins, isoflavones, and lignans, into more bioavailable aglycone forms and microbial metabolites, including urolithins, equol, and enterolignans. These metabolites may exhibit enhanced anti-inflammatory, antioxidant, and estrogen receptor-modulating activities, with potential implications for adipose tissue function and neuroinflammatory tone [300]. In addition, the microbial fermentation of dietary fiber and resistant starch in the colon leads to the production of short-chain fatty acids (acetate, propionate, and butyrate). These fatty acids act as signaling molecules via free fatty acid receptors expressed on enteroendocrine cells, adipocytes, and immune cells, stimulating the secretion of GLP-1 and PYY, modulating adipose tissue lipolysis, and exerting anti-inflammatory effects on colonic and systemic immune cells [301].

### 8.3. Technological Innovations for Anti-Obesity Functional Foods

Applying mechanistic insights into the adipose tissue-central nervous system axis to effective dietary interventions requires not only the identification of bioactive compounds with proven metabolic targets, but also the development of technological platforms capable of overcoming the inherent limitations of food formulation, including poor bioavailability, chemical instability, organoleptic incompatibility, gastrointestinal degradation, limited tissue targeting, and uncontrolled release. Since the biological activity of many nutraceuticals depends on their ability to reach metabolically active tissues at effective concentrations, food technologies have emerged as enabling tools to enhance the delivery of bioactive compounds to adipose tissue and the CNS. By improving absorption, stability, release kinetics, and tissue-specific distribution, these technologies may potentiate the modulation of adipokine signaling, neuroinflammation, nutrient sensing, and gut-derived metabolic pathways, thereby strengthening the mechanistic interaction along the adipose tissue–CNS axis. Accordingly, the following subsections discuss emerging food technologies not merely as formulation strategies, but as platforms designed to improve the biological efficacy of nutritional interventions targeting adipose tissue–brain communication.

#### 8.3.1. Nanoencapsulation and Microencapsulation Technologies

Encapsulation technologies are based on the inclusion of bioactive compounds within protective carrier matrices that can shield them from environmental degradation, modulate their release kinetics, and enhance their gastrointestinal stability and intestinal absorption. The physical scale of the delivery system, nano (1–1000 nm) or micro (1–1000 μm), determines its interaction with gastrointestinal physiology, mucosal penetration, mechanisms of absorption by enterocytes, and systemic distribution [302].

Nanoemulsions have been widely investigated for the delivery of lipophilic bioactive compounds, including omega-3 polyunsaturated fatty acids, curcumin, resveratrol, quercetin, and fat-soluble vitamins. Although nanoemulsions are thermodynamically unstable, they can be kinetically stable when properly formulated, and their small droplet size provides important biophysical advantages. In particular, the increased interfacial surface area facilitates faster and more complete lipolysis by pancreatic lipase, accelerating the transfer of lipophilic compounds into mixed micelles and improving their intestinal absorption [303]. In the context of neuroinflammation and the adipose tissue–CNS axis, DHA and EPA administered via nanoemulsions have demonstrated significantly improved bioavailability and brain uptake compared to equivalent doses in the form of conventional triglycerides [304] Similarly, curcumin nanoemulsions have demonstrated significantly greater anti-neuroinflammatory efficacy than native curcumin, overcoming the compound’s limitations, such as poor water solubility, rapid metabolic conjugation, and limited permeability of the blood–brain barrier [305].

Liposomes, vesicles composed of one or more phospholipid bilayers that enclose an aqueous core, offer the advantage of being able to accommodate both hydrophilic compounds within their aqueous core and lipophilic compounds within their phospholipid bilayer. Liposomes used in the food industry, prepared with lecithin, phosphatidylcholine, and other phospholipids, have been used to encapsulate polyphenols, vitamins, and bioactive peptides, improving their gastrointestinal stability and mucosal permeability [306]. Of particular relevance to metabolic health, liposomal delivery of resveratrol has been reported to increase its systemic bioavailability compared with free resveratrol, potentially enhancing biological pathways related to SIRT1 activation, NF-κB inhibition, and adiponectin regulation in adipose tissue [307]. Liposomes containing phosphatidylserine also interact with the lipid composition of the hypothalamic membrane, modulating receptor localization and signal transduction related to insulin and leptin sensitivity [308].

Solid lipid nanoparticles (SLNs) and nanostructured lipid carriers (NLCs) represent an evolution of lipid-based delivery systems, utilizing solid or partially solid lipid matrices that provide greater chemical protection for compounds such as omega-3 fatty acids, coenzyme Q10, and fat-soluble vitamins, which are prone to oxidation compared to liquid emulsion systems. The solid state of the lipid matrix slows down lipid oxidation and delays the release of the encapsulated compounds during gastrointestinal transit, allowing for a more prolonged and controlled release to the intestinal epithelium [309].

Biopolymer-based delivery systems, including protein nanoparticles, polysaccharide hydrogels, and complex coacervates formed through electrostatic interactions between biopolymers, offer the advantage of being prepared without the use of organic solvents and enabling pH-sensitive or enzyme-triggered release profiles [310]. For example, coacervates of whey protein and pectin can protect polyphenols from degradation by stomach acid, releasing them preferentially into the alkaline intestinal environment and maximizing their bioavailability at the site of absorption [311]. Chitosan nanoparticles, by leveraging the mucoadhesive properties of this cationic polysaccharide, prolong the retention time in the intestinal mucosal layer and enhance the absorption of the encapsulated compounds through both transcellular and paracellular pathways [312].

Resistant starch microparticles and inulin-based matrices have been designed to deliver butyrate, or its precursors, to the colon, where it exerts local anti-inflammatory effects on colonocytes, improves the integrity of the intestinal barrier, and promotes the secretion of GLP-1 and PYY, modulating appetite and energy balance through gut–brain axis mechanisms [313]. Encapsulated long-chain fatty acids and fermentable fibers formulated for ileal release have demonstrated a greater postprandial satiety response and reduced energy intake in human and animal studies, supporting their potential to modulate appetite via the gut–brain axis [271]. Pectin-based hydrogel microspheres, which incorporate omega-3 fatty acids or polyphenols, have been shown to release these compounds selectively into the colonic environment through the hydrolysis of pectin by microbial pectinases, thereby amplifying the anti-inflammatory effects of these compounds at the mucosal level [314].

#### 8.3.2. Precision Fermentation

Precision fermentation represents a rapidly expanding biotechnological frontier with significant implications for the development of functional foods targeting obesity-related metabolic dysfunction and gut–brain–adipose tissue signaling. By programming microbial cell factories with heterologous biosynthetic pathways, precision fermentation enables the scalable production of bioactive compounds that are rare, unstable, or difficult to extract from natural sources. These include specific omega-3 fatty acids, such as DHA from non-fish sources, bioactive peptides with defined receptor-related activities, and plant-derived polyphenols such as resveratrol and pterostilbene [315,316].

Beyond the production of purified bioactive ingredients, synthetic biology also enables the development of engineered microbial systems designed to deliver biologically active molecules directly within the gastrointestinal tract. Recombinant Lactobacillus strains engineered to express and secrete glucagon-like peptide-1 (GLP-1) or its precursors have shown improvements in glucose homeostasis, insulin secretion, and body-weight-related outcomes in experimental models [317]. Similarly, it has been shown that engineered strains that produce N-acyl phosphatidylethanolamines, precursors of N-acyl ethanolamine, a lipid mediator that promotes satiety, reduce food intake and adiposity in mice by acting through the signaling pathways of hypothalamic cannabinoid receptors and peroxisome proliferator-activated receptors [318]. Synthetic biology approaches may also make it possible to engineer the composition of the gut microbiota itself by designing specific microbial consortia with complementary metabolic functions optimized for the production of short-chain fatty acids, the transformation of bile acids, and the modulation of inflammatory responses, potentially offering a more robust and sustainable approach to metabolic reprogramming than conventional probiotic supplementation [319].

#### 8.3.3. Three-Dimensional Food Printing

Three-dimensional food printing represents a paradigm change in the conception of food as a tool for precision delivery, enabling simultaneous control over the composition of macronutrients, the content of micronutrients, the concentration of bioactive compounds, the structural properties, and the matrix architecture within the product [320,321]. Three-dimensional printing offers the ability to design food matrices with precisely defined porosity, density gradients, and spatial compartmentalization of nutrients, allowing for the independent optimization of parameters such as masticatory resistance, gastric emptying rate, intestinal transit time, and nutrient release at specific sites—all factors that influence postprandial secretion of gut hormones, satiety signaling, and metabolic responses along the adipose tissue–CNS axis [322].

Extrusion-based 3D printing systems, which use hydrogel inks composed of food-grade biopolymers such as alginate, gelatin, pectin, and methylcellulose, can incorporate encapsulated bioactive compounds, including omega-3 fatty acids, polyphenols, probiotics, and bioactive peptides, within structurally defined matrices that protect them during printing and subsequent gastrointestinal processing [323]. The ability to print personalized portions and macronutrient ratios tailored to individual metabolic phenotypes opens the door to personalized functional dietary interventions, in line with the principles of precision nutrition [324]. In addition, 3D printing enables the co-deposition of prebiotic fibers in close proximity to probiotic microorganisms within the same food matrix, creating symbiotic combinations that offer greater colonization efficiency and metabolic activity compared to conventional supplementation formats [320].

Despite these promising findings, most of the evidence supporting the effects of bioactive compounds and advanced delivery technologies on the adipose tissue–CNS axis derives from in vitro and animal studies. Therefore, their clinical efficacy, long-term safety, optimal dosage, and capacity to improve metabolic and neurological outcomes in humans remain to be established through well-designed controlled clinical trials. Accordingly, the available evidence should not be interpreted as demonstrating definitive causal or therapeutic effects in humans.

## 9. Conclusions

The adipose tissue–CNS axis represents a cornerstone of systemic bioenergetic homeostasis. Current evidence supports an integrated model in which pathological adipose tissue expansion contributes to peripheral lipotoxicity and inflammatory signaling that may propagate to the CNS, resulting in neuroinflammation and alterations in the brain metabolome, including impaired NAD^+^ metabolism and altered branched-chain amino acid (BCAA) homeostasis. Among the most promising molecular targets identified in this review are the lipid receptor FFAR4/GPR120, which mediates hypothalamic anti-inflammatory responses to polyunsaturated fatty acids; the SCFA receptors FFAR2/GPR43 and FFAR3/GPR41, involved in microbiota–gut–brain communication; and central leptin-signaling pathways mediated by BDNF in the paraventricular nucleus. However, the clinical translation of these findings presents major challenges, including the difficulty of selectively targeting CNS circuits without inducing unwanted peripheral effects and the limited transport of many bioactive compounds across the blood–brain barrier. Nevertheless, most mechanistic evidence derives from experimental models, and validation of these pathways in humans remains a major priority. Future research should clarify the causal relationships within this bidirectional network through large-scale controlled clinical trials integrating metabolic, neuroimaging, and multiomic approaches. Such efforts will be essential to validate therapeutic targets and determine whether precision nutrition and emerging food technologies can effectively restore adipose tissue–CNS homeostasis and improve metabolic resilience in humans.

## Figures and Tables

**Table 1:** Key Studies on Dietary Modulation of the Adipose Tissue–CNS Axis: Mechanisms and Outcomes.

Authors (Year)	Study Type/Model	Dietary Exposure	Main Mechanism on the CNS–Adipose Tissue Axis	Main Outcomes	References
Estrada & Contreras (2019)	Review (mechanistic)	High SFA/sugars; Western-type diets	Systemic low-grade inflammation → microglial activation; disruption of immune–CNS homeostasis	↑ Risk of neurological disorders in obesogenic contexts	[236]
Puente-Ruiz & Jais (2022)	Review	—	Bidirectional adipokine/cytokine signaling adipose ↔ CNS; sympathetic regulation of depots	Links obesity to metabolic dysfunction and neurodegeneration	[235]
Bruce-Keller et al. (2009)	Review (human + animal)	Diet-induced obesity	Oxidative stress & inflammation; impaired neuronal plasticity	↑ CNS vulnerability, cognitive decline	[237]
Ryan, Woods & Seeley (2012)	Physiological review	Palatable high-fat diets	Elevation of “defended adiposity” via hypothalamic circuits (homeostatic set-point)	Greater difficulty maintaining weight loss	[241]
Kaiyala et al. (2000)	Experimental (dog model)	High-fat diet	↓ Insulin transport into CSF/CNS → weaker negative feedback on adiposity	Increased adiposity and weight gain with reduced CNS insulin delivery	[242]
Jamar, Ribeiro & Pisani (2021)	Review	High-fat/high-sugar diets	Microbiota–gut–brain axis dysbiosis; microglial activation; hypothalamic inflammation	Obesity, metabolic syndrome, neuroinflammation	[239]
Yu et al. (2022)	Review	Diet-related dysbiosis	Crosstalk adipose tissue ↔ microbiota–gut–brain via microbial metabolites & immune cells	Systemic dysmetabolism impacting CNS signaling	[244]
Custers & Kiliaan (2022)	Review	Dietary lipids	BBB transport & brain lipid remodeling; altered lipid metabolism in neural tissue	Contribution to AD, PD, stroke mechanisms	[243]
Solas et al. (2017)	Review	Obesity/dietary patterns	Peripheral cytokines & vagal signaling; gut–brain–inflammation triad	Cognitive decline pathways and potential pharmacological targets	[240]
Frausto et al. (2021)	Review	Microbiota modulation	SCFAs, bile acids, Trp-derivatives → microglia/astroglia and amyloid pathways	Mechanistic links to Alzheimer’s disease	[245]
Godos et al. (2020)	Review	Antioxidant/omega-3-rich diets	↓ Oxidative stress & neuroinflammation; neurotransmission & neurogenesis modulation	↓ Depression/anxiety risk; mental health support	[247]
Granero & Guillazo-Blanch (2025)	Editorial (Special Issue)	Mediterranean/MIND dietary patterns	↓ Inflammation; ↑ neuroplasticity and cognitive resilience	Association with ↓ risk of cognitive decline	[246]
Léon, Nadjar & Quarta (2021)	Perspective/Mechanistic review	Hypercaloric/high-fat diets	Maladaptive microglia–neuron crosstalk in hypothalamus; cytokines & fuel-sensing	Hypothalamic dysfunction → energy imbalance & obesity progression	[238]

Representative studies illustrating how dietary patterns and nutritional exposures influence inflammation, hypothalamic function, adipose tissue signaling, gut microbiota interactions, and systemic metabolic homeostasis. Abbreviations: ↑, increased; ↓, decreased; →, leads to; ↔, bidirectional interaction; AD, Alzheimer’s disease; BBB, blood–brain barrier; CNS, central nervous system; CSF, cerebrospinal fluid; MIND, Mediterranean-DASH Intervention for Neurodegenerative Delay; PD, Parkinson’s disease; SCFAs, short-chain fatty acids; SFA, saturated fatty acids; Trp, tryptophan.

**Table 2:** Effects of dietary interventions on inflammation and appetite regulation along the adipose tissue–CNS axis.

Authors (Year)	Study Type/Model	Dietary Exposure	Main Mechanism on the CNS–Adipose Tissue Axis	Main Outcomes	References
Adepoju et al. (2026)	RCT (humans, obesity)	Almond consumption	↓ systemic inflammation (cytokines) → indirect CNS signaling	↓ IL-6, TNF-α; no effect on appetite	[248]
Åberg et al. (2025)	RCT (humans)	Wholegrain rye vs. refined wheat	Modulation of gut hormones (ghrelin, incretins)	↓ ghrelin (postprandial), improved glycemia	[251]
Zhang et al. (2024)	RCT crossover (humans)	Eating frequency	No significant modulation of appetite/inflammation axis	No changes in ghrelin, leptin, CRP	[250]
Sanchez et al. (2024)	Animal study (mice)	High-fat diet (ω6/ω3 ratio)	Neuroinflammation (hypothalamus, hippocampus)	↑ neuroinflammation, behavioral changes	[258]
Vujović et al. (2022)	RCT crossover (humans)	Meal timing (late vs. early eating)	Hormonal dysregulation (ghrelin/leptin) + adipose gene expression	↑ hunger, ↓ energy expenditure	[252]
Reginato et al. (2021)	Systematic review	High-fat diet/fatty acids	Ceramide accumulation → hypothalamic inflammation → leptin resistance	Impaired energy balance regulation	[257]
Reimer et al. (2017)	RCT (humans)	Prebiotics + whey protein	Gut microbiota modulation → appetite signaling	↓ hunger, altered microbiota	[254]
Juanola-Falgarona et al. (2014)	RCT (humans)	Low vs. high glycemic index diets	Metabolic signaling (insulin sensitivity)	↓ weight, no strong effect on appetite/inflammation	[253]
Poulsen et al. (2014)	RCT crossover (humans)	Advanced glycation end products (AGEs)	Acute hormonal modulation (ghrelin)	↑ ghrelin response; limited inflammation effects	[256]
Nilsson et al. (2013)	RCT crossover (humans)	Brown beans (fiber-rich meal)	SCFA production → gut–brain signaling	↑ satiety hormones, ↓ IL-6	[255]
Sofer et al. (2011)	RCT (humans)	Carbohydrate timing (evening intake)	Hormonal modulation (leptin, adiponectin)	↓ hunger, ↓ inflammatory markers	[249]

Dietary interventions influence inflammation and appetite regulation through multiple mechanisms along the adipose tissue–central nervous system axis. Evidence from randomized controlled trials, animal studies, and mechanistic reviews highlights both peripheral (e.g., cytokines, gut microbiota) and central (e.g., hypothalamic inflammation, hormonal signaling) pathways. Abbreviations: ↑, increased; ↓, decreased; →, leads to; ω, omega; AGEs, advanced glycation end products; CNS, central nervous system; CRP, C-reactive protein; IL-6, interleukin-6; RCT, randomized controlled trial; SCFA, short-chain fatty acid; TNF-α, tumor necrosis factor-alpha.

**Table 3:** Effects of nutritional strategies on metabolic health and the CNS–adipose tissue axis.

Authors (Year)	Study Type/Model	Dietary Exposure	Proposed Mechanisms on the CNS–Adipose Tissue Axis	Main Outcomes	References
Asoudeh et al. (2023)	RCT, adolescents with MetS	Mediterranean diet	↓ systemic inflammation may influence adipose–CNS signaling	↓ IL-6, hs-CRP, improved HOMA-IR and lipid profile	[259]
Vitale et al. (2020)	RCT, overweight/obese adults	Mediterranean-like diet	↑ SCFAs may contribute to gut–brain signaling and metabolic regulation	↑ insulin sensitivity, ↓ postprandial glucose, microbiota shifts	[260]
Ulven et al. (2019)	RCT, MetS subjects	Nordic diet	Modulation of inflammatory gene expression may affect systemic–central signaling pathways	↓ TNFRSF1A, ↑ RELA expression	[261]
Calvo-Malvar et al. (2021)	Cluster RCT, families	Atlantic diet	Improved dietary pattern may indirectly influence adiposity-related signaling to CNS	↓ body weight, ↓ total cholesterol	[262]
Vetrani et al. (2016)	RCT, MetS subjects	Whole-grain diet	↑ SCFAs (propionate) may modulate gut–brain axis and insulin signaling	↑ propionate, ↓ postprandial insulin	[263]
Zhang et al. (2022)	RCT, overweight/obese adults	Avocado (MUFA + fiber)	Changes in lipid profile and inflammation may influence central metabolic regulation	↓ CRP; modest effects on glycemic control	[264]
Pett et al. (2025)	RCT, overweight/obese adults	Mango intake	Antioxidant pathways (e.g., Nrf2) may indirectly affect metabolic and central signaling	↑ insulin sensitivity (HOMA-IR), no significant change in inflammation	[265]
Palacios et al. (2019)	RCT, prediabetes adults	Almonds vs. CHO foods	Improved nutrient composition may have limited impact on CNS–adipose signaling	No significant effects on insulin sensitivity; improved nutrient profile	[266]

Randomized controlled trials evaluating dietary patterns and food-based interventions and their effects on metabolic health, with proposed mechanisms potentially linking inflammation, gut microbiota, and CNS–adipose tissue signaling. Abbreviations: ↑, increased; ↓, decreased; CHO, carbohydrates; CNS, central nervous system; CRP, C-reactive protein; HOMA-IR, Homeostatic Model Assessment of Insulin Resistance; hs-CRP, high-sensitivity C-reactive protein; IL-6, interleukin-6; MetS, metabolic syndrome; MUFA, monounsaturated fatty acids; Nrf2, nuclear factor erythroid 2-related factor 2; RCT, randomized controlled trial; SCFAs, short-chain fatty acids; TNFRSF1A, tumor necrosis factor receptor superfamily member 1A.

**Table 4:** Emerging technologies for studying and translating the adipose tissue–CNS axis.

Technology	Main Application	Strengths	Limitations	Translational Potential
Single-cell RNA sequencing	Identification of adipocyte, immune and hypothalamic cell populations	High cellular resolution; identifies rare cell populations	Tissue dissociation, loss of spatial information, expensive	Biomarker discovery; precision medicine
Spatial transcriptomics	Maps gene expression within adipose tissue and hypothalamus	Preserves tissue architecture; identifies cell–cell interactions	High cost; limited availability	Identification of disease-specific niches
Single-cell multiomics	Integrates transcriptome, chromatin accessibility and epigenetics	Comprehensive molecular characterization	Computational complexity	Patient stratification
Extracellular vesicle profiling	Characterization of adipose-derived EVs reaching the CNS	Minimally invasive biomarkers	Isolation and standardization remain challenging	Early diagnosis; therapeutic monitoring
Advanced neuroimaging (MRI, PET)	Assessment of hypothalamic inflammation and brain connectivity	Applicable in humans; longitudinal studies	Indirect markers; expensive	High
Metabolomics/Lipidomics	Identification of circulating metabolic mediators	High-throughput; identifies metabolic signatures	Biological variability	Biomarker discovery
Chemogenetics	Functional manipulation of neuronal circuits	Demonstrates causality	Animal models only	Low (preclinical)
Optogenetics	Precise temporal control of neural circuits	Excellent mechanistic tool	Animal models only	Low
Organoids/Organ-on-chip	Human tissue modeling	Human-derived models; personalized approaches	Limited physiological complexity	Medium
Artificial intelligence integration	Integration of multiomics and imaging	Predictive models; precision nutrition	Requires large datasets	Very high

Overview of current technological approaches, including single-cell and spatial omics, neuroimaging, extracellular vesicle profiling, chemogenetics, organ-on-chip systems, and artificial intelligence-based integration. Strengths, limitations, and translational implications are summarized.

## Data Availability

Data are available from the authors upon reasonable request.

## Abbreviations

The following abbreviations are used in this manuscript:

ACC1	Acetyl-CoA carboxylase 1
ACLY	ATP citrate lyase
Acrp30	Adipocyte complement-related protein of 30 kDa
AD	Alzheimer’s disease
AdipoQ	Adiponectin
AdipoR1	Adiponectin receptor 1
AdipoR2	Adiponectin receptor 2
ADSCs	Adipose-derived stem cells
AGEs	Advanced glycation end products
AGPAT	1-Acylglycerol-3-phosphate O-acyltransferase
AgRP	Agouti-related peptide
Akt	Protein kinase B
AMPK	AMP-activated protein kinase
APCs	Adipose progenitor cells
APJ	Apelin receptor
ARC	Arcuate nucleus
ATGL	Adipose triglyceride lipase
ATMs	Adipose tissue macrophages
BAMs	Border-associated macrophages
BAT	Brown adipose tissue
BBB	Blood–brain barrier
BCAAs	Branched-chain amino acids
BDNF	Brain-derived neurotrophic factor
cAMP	Cyclic adenosine monophosphate
CAT	Catalase
CD36	Cluster of differentiation 36
CerS6	Ceramide synthase 6
CHO	Carbohydrate
ChREBP	Carbohydrate-responsive element-binding protein
CNS	Central nervous system
CoA	Coenzyme A
COX	Cyclooxygenase
COX-2	Cyclooxygenase-2
CRP	C-reactive protein
CSF	Cerebrospinal fluid
DAGs	Diacylglycerols
DGAT	Diacylglycerol acyltransferase
DHA	Docosahexaenoic acid
DNA	Deoxyribonucleic acid
DNL	De novo lipogenesis
DPP-4	Dipeptidyl peptidase-4
DRG	Dorsal root ganglia
DRP1	Dynamin-related protein 1
ECM	Extracellular matrix
EPA	Eicosapentaenoic acid
ER	Endoplasmic reticulum
EVs	Extracellular vesicles
FABP4	Fatty acid-binding protein 4
FASN	Fatty acid synthase
FATP1	Fatty acid transport protein 1
FFAR1/GPR40	Free fatty acid receptor 1/G protein-coupled receptor 40
FFAR2/GPR43	Free fatty acid receptor 2/G protein-coupled receptor 43
FFAR3/GPR41	Free fatty acid receptor 3/G protein-coupled receptor 41
FFAR4/GPR120	Free fatty acid receptor 4/G protein-coupled receptor 120
FFARs	Free fatty acid receptors
FFAs	Free fatty acids
FIS1	Mitochondrial fission 1 protein
FUNDC1	FUN14 domain-containing protein 1
FXR	Farnesoid X receptor
GIP	Glucose-dependent insulinotropic polypeptide
GLP-1	Glucagon-like peptide-1
GLUT1	Glucose transporter 1
GLUT4	Glucose transporter 4
GPAT	Glycerol-3-phosphate acyltransferase
GPCRs	G protein-coupled receptors
GPx	Glutathione peroxidase
Gsα	Stimulatory G protein alpha subunit
HFD	High-fat diet
HIFs	Hypoxia-inducible factors
HOMA-IR	Homeostatic model assessment of insulin resistance
HPA	Hypothalamic–pituitary–adrenal axis
HPP	High-pressure processing
hs-CRP	High-sensitivity C-reactive protein
HSL	Hormone-sensitive lipase
IFN-γ	Interferon gamma
IL-1β	Interleukin-1 beta
IL-6	Interleukin-6
ILC2s	Group 2 innate lymphoid cells
JNK	c-Jun N-terminal kinase
LDL	Low-density lipoprotein
lnc-BATE1	Brown adipose tissue-enriched long non-coding RNA 1
lnc-MALAT1	Metastasis-associated lung adenocarcinoma transcript 1
lncRNAs	Long non-coding RNAs
LOX	Lipoxygenase
LPA	Lysophosphatidic acid
LPAR1–6	Lysophosphatidic acid receptors 1–6
LXRs	Liver X receptors
M1	Classically activated macrophage phenotype
M2	Alternatively activated macrophage phenotype
MBH	Mediobasal hypothalamus
MCH	Melanin-concentrating hormone
mDIC	Mitochondrial dicarboxylate carrier
MetS	Metabolic syndrome
MFF	Mitochondrial fission factor
MFN1	Mitofusin 1
MFN2	Mitofusin 2
MiD49	Mitochondrial dynamics protein of 49 kDa
MiD51	Mitochondrial dynamics protein of 51 kDa
MIND	Mediterranean–Dietary Approaches to Stop Hypertension Intervention for Neurodegenerative Delay
miR-155	MicroRNA-155
miR-27a	MicroRNA-27a
miR-34a	MicroRNA-34a
miR-9-3p	MicroRNA-9-3p
miRNAs	MicroRNAs
MRI	Magnetic resonance imaging
mRNAs	Messenger RNAs
MRP4	Multidrug resistance-associated protein 4
mtDNA	Mitochondrial DNA
mTOR	Mechanistic target of rapamycin
mTORC1	Mechanistic target of rapamycin complex 1
MUFA	Monounsaturated fatty acid
NADPH	Nicotinamide adenine dinucleotide phosphate
NAD^+^	Nicotinamide adenine dinucleotide
NE	Norepinephrine
NEIL1	Nei-like DNA glycosylase 1
NF-κB	Nuclear factor kappa B
NLCs	Nanostructured lipid carriers
NOTCH2	Notch receptor 2
NPY	Neuropeptide Y
Nrf2	Nuclear factor erythroid 2-related factor 2
Nscl-2	Neuronal stem cell leukemia 2
OAT3	Organic anion transporter 3
OGG1	8-Oxoguanine DNA glycosylase 1
OMA1	OMA1 zinc metallopeptidase
OPA1	Optic atrophy protein 1
P450	Cytochrome P450
PAI-1	Plasminogen activator inhibitor-1
PARPs	Poly(ADP-ribose) polymerases
PD	Parkinson’s disease
PET	Positron emission tomography
PGC-1α	Peroxisome proliferator-activated receptor gamma coactivator 1-alpha
PGE2	Prostaglandin E2
PKA	Protein kinase A
PKC	Protein kinase C
PKCζ	Protein kinase C zeta
PLIN1	Perilipin 1
PNPLA2	Patatin-like phospholipase domain-containing protein 2
POMC	Pro-opiomelanocortin
PON2	Paraoxonase 2
PP2A	Protein phosphatase 2A
PPARs	Peroxisome proliferator-activated receptors
PPARγ	Peroxisome proliferator-activated receptor gamma
PRDM16	PR/SET domain 16
Prx3	Peroxiredoxin 3
PUFAs	Polyunsaturated fatty acids
PVH	Paraventricular nucleus of the hypothalamus
PYY	Peptide YY
RAGE	Receptor for advanced glycation end products
RBP4	Retinol-binding protein 4
RCTs	Randomized controlled trials
RELA	RELA proto-oncogene, NF-κB subunit
RELM	Resistin-like molecule
Rho	Ras homolog family GTPase
RNA	Ribonucleic acid
ROCK	Rho-associated coiled-coil-containing protein kinase
ROS	Reactive oxygen species
S1P	Sphingosine-1-phosphate
S1PR1–S1PR5	Sphingosine-1-phosphate receptors 1–5
SAM	S-adenosylmethionine
SAT	Subcutaneous adipose tissue
SCFAs	Short-chain fatty acids
SCN	Suprachiasmatic nucleus
SFAs	Saturated fatty acids
SIRT1	Sirtuin 1
SIRT6	Sirtuin 6
SLNs	Solid lipid nanoparticles
SNS	Sympathetic nervous system
SOD	Superoxide dismutase
SPT	Serine palmitoyltransferase
STAT3	Signal transducer and activator of transcription 3
TAGs	Triacylglycerols
TCA	Tricarboxylic acid cycle
TGR5	Takeda G protein-coupled receptor 5
TH	Tyrosine hydroxylase
TLR4	Toll-like receptor 4
TNF-α	Tumor necrosis factor alpha
TNFRSF1A	Tumor necrosis factor receptor superfamily member 1A
UCP1	Uncoupling protein 1
VAT	Visceral adipose tissue
VLDL	Very-low-density lipoprotein
WAT	White adipose tissue
WNT	Wingless-related integration site
αMSH	Alpha-melanocyte-stimulating hormone
βARs	Beta-adrenergic receptors
